# Translational development of ABCB5^+^ dermal mesenchymal stem cells for therapeutic induction of angiogenesis in non-healing diabetic foot ulcers

**DOI:** 10.1186/s13287-022-03156-9

**Published:** 2022-09-05

**Authors:** Andreas Kerstan, Kathrin Dieter, Elke Niebergall-Roth, Sabrina Klingele, Michael Jünger, Christoph Hasslacher, Georg Daeschlein, Lutz Stemler, Ulrich Meyer-Pannwitt, Kristin Schubert, Gerhard Klausmann, Titus Raab, Matthias Goebeler, Korinna Kraft, Jasmina Esterlechner, Hannes M. Schröder, Samar Sadeghi, Seda Ballikaya, Martin Gasser, Ana M. Waaga-Gasser, George F. Murphy, Dennis P. Orgill, Natasha Y. Frank, Christoph Ganss, Karin Scharffetter-Kochanek, Markus H. Frank, Mark A. Kluth

**Affiliations:** 1 Department of Dermatology, Venereology and Allergology, University Hospital Würzburg, Würzburg, Germany; 2 RHEACELL GmbH & Co. KG, Heidelberg, Germany; 3 TICEBA GmbH, Im Neuenheimer Feld 517, 69120 Heidelberg, Germany; 4 Department of Dermatology, University Hospital Greifswald, Greifswald, Germany; 5 Clinical Study Center St. Josefskrankenhaus, Heidelberg, Germany; 6 Diabetologikum DDG Ludwigshafen, Ludwigshafen, Germany; 7 Department of Clinical Research and Development, MARE Clinic, Kiel, Germany; 8 medamed GmbH, Leipzig, Germany; 9 Studienzentrum Aschaffenburg, Aschaffenburg, Germany; 10 Diabetologikum Raab, Kassel, Germany; 11 Department of Surgery, University Hospital Würzburg, Würzburg, Germany; 12 Division of Renal (Kidney) Medicine, Brigham and Women’s Hospital, Harvard Medical School, Boston, MA USA; 13 Department of Dermatology, Brigham and Women’s Hospital, Harvard Medical School, Boston, MA USA; 14 Division of Plastic Surgery, Brigham and Women’s Hospital, Harvard Medical School, Boston, MA USA; 15 Department of Medicine, VA Boston Healthcare System, Boston, MA USA; 16 Division of Genetics, Brigham and Women’s Hospital, Harvard Medical School, Boston, MA USA; 17 Transplant Research Program, Boston Children’s Hospital, Harvard Medical School, Boston, MA USA; 18 Harvard Stem Cell Institute, Harvard University, Cambridge, MA USA; 19 Department of Dermatology and Allergic Diseases, University Hospital, Ulm, Germany; 20 School of Medical and Health Sciences, Edith Cowan University, Perth, Australia; 21 Present Address: Clinic of Dermatology, Immunology and Allergology, Medical University Brandenburg “Theodor Fontane” Medical Center Dessau, Dessau, Germany

**Keywords:** ABCB5, Advanced-therapy medicinal product, Angiogenesis, Chronic wound, Diabetic foot ulcer, Mesenchymal stem cells, Wound healing

## Abstract

**Background:**

While rapid healing of diabetic foot ulcers (DFUs) is highly desirable to avoid infections, amputations and life-threatening complications, DFUs often respond poorly to standard treatment. GMP-manufactured skin-derived ABCB5^+^ mesenchymal stem cells (MSCs) might provide a new adjunctive DFU treatment, based on their remarkable skin wound homing and engraftment potential, their ability to adaptively respond to inflammatory signals, and their wound healing-promoting efficacy in mouse wound models and human chronic venous ulcers.

**Methods:**

The angiogenic potential of ABCB5^+^ MSCs was characterized with respect to angiogenic factor expression at the mRNA and protein level, *in vitro* endothelial trans-differentiation and tube formation potential, and perfusion-restoring capacity in a mouse hindlimb ischemia model. Finally, the efficacy and safety of ABCB5^+^ MSCs for topical adjunctive treatment of chronic, standard therapy-refractory, neuropathic plantar DFUs were assessed in an open-label single-arm clinical trial.

**Results:**

Hypoxic incubation of ABCB5^+^ MSCs led to posttranslational stabilization of the hypoxia-inducible transcription factor 1*α* (HIF-1*α*) and upregulation of HIF-1*α* mRNA levels. HIF-1*α* pathway activation was accompanied by upregulation of vascular endothelial growth factor (VEGF) transcription and increase in VEGF protein secretion. Upon culture in growth factor-supplemented medium, ABCB5^+^ MSCs expressed the endothelial-lineage marker CD31, and after seeding on gel matrix, ABCB5^+^ MSCs demonstrated formation of capillary-like structures comparable with human umbilical vein endothelial cells. Intramuscularly injected ABCB5^+^ MSCs to mice with surgically induced hindlimb ischemia accelerated perfusion recovery as measured by laser Doppler blood perfusion imaging and enhanced capillary proliferation and vascularization in the ischemic muscles. Adjunctive topical application of ABCB5^+^ MSCs onto therapy-refractory DFUs elicited median wound surface area reductions from baseline of 59% (full analysis set, *n* = 23), 64% (per-protocol set, *n* = 20) and 67% (subgroup of responders, *n* = 17) at week 12, while no treatment-related adverse events were observed.

**Conclusions:**

The present observations identify GMP-manufactured ABCB5^+^ dermal MSCs as a potential, safe candidate for adjunctive therapy of otherwise incurable DFUs and justify the conduct of a larger, randomized controlled trial to validate the clinical efficacy.

*Trial registration*: ClinicalTrials.gov, NCT03267784, Registered 30 August 2017, https://clinicaltrials.gov/ct2/show/NCT03267784

**Supplementary Information:**

The online version contains supplementary material available at 10.1186/s13287-022-03156-9.

## Background

Diabetic foot ulcers (DFUs) are among the most common and potentially serious complications of diabetes mellitus, with an estimated 19% to 34% of diabetes patients developing a DFU during their lifetimes [1]. Around 40% of patients who have developed a DFU die within 5 years [2–4]. While a significant proportion of the mortality rates can be attributed to fatal cardio-vascular complications of diabetes [5–7], the ulcer contributes independently to mortality due to inflammatory sequelae [4, 8, 9]. Specifically, more than half of DFUs become infected [10], with roughly 20% to 50% of moderate-to-severe diabetic foot infections potentially leading to some grade of lower extremity amputation [1, 11–14]. Many patients who underwent a DFU-related amputation have a poor quality of life and a high risk of premature death [15].

While rapid healing is highly desirable to avoid infections, amputations and life-threatening complications [16–18], DFUs often respond poorly to standard treatment. Reported healing failure rates range from roughly 40% to 80% at 12 weeks and still from 15 to 70% at 1 year of treatment (Additional file 1: Table S1). Current treatment guidelines advocate to consider adjunctive therapy options for DFUs that have not achieved a 50% area reduction within 4 weeks [19–21] or failed to heal after 4–6 weeks [22] of standard wound care. There are an increasing number of therapeutic efforts to speed the healing of DFUs, and the literature surrounding their use is evolving [23].

From a pathophysiologic perspective, dysfunctional wound healing in diabetes is closely linked to insufficient angiogenesis [24], caused by a chronic inflammatory disposition in concert with impaired cellular responses to tissue hypoxia [25–27]. A sustained, interleukin (IL)-1β-driven prevalence of pro-inflammatory M1 macrophages associated with defective transition to reparative, granulation-promoting M2 macrophages [28–33] and an impaired activation of the hypoxia-inducible transcription factor 1*α* (HIF-1*α*) pathway by local fibroblasts and endothelial cells [34–36] leading to deficient HIF-1*α*-dependent upregulation of multiple angiogenic factors including vascular endothelial growth factor (VEGF) [34–38] ultimately result in a decreased amount of nascent microvasculature [39, 40].

In the light of a complex pathophysiology, mesenchymal stem cells (MSCs), derived from various sources, including adipose tissue, bone marrow, peripheral blood and umbilical cord, have been extensively investigated and considered a promising approach to adjunctive DFU treatment [41–43], owing to their remarkable ability to adaptively respond to signals associated with tissue injury and inflammation by providing paracrine signals which alter the wound environment toward a pro-healing state or even directly participate in wound regeneration [43, 44]. MSCs, among others particularly adipose tissue-derived MSCs or stromal vascular fraction cells, have been safely and successfully used alone or combined with dermal substitutes or autologous growth factors such as platelet-rich plasma to treat skin wounds and scars of various etiologies including diabetic and vascular ulcers, burn wounds, post-traumatic wounds [45, 46]. Owing to a wide range of immunomodulatory capacities involving direct interactions with immune cells as well as various paracrine pathways [47], they are considered attractive candidates for the treatment of local and systemic inflammatory conditions including even COVID-19 [48]. However, most of the investigated MSC therapies have not progressed beyond early-stage clinical trials [49], and translation of an MSC-based approach for the treatment of otherwise non-healing DFUs that is readily (off the shelf) available and does not require an elaborate surgical procedure into clinical practice has not yet been achieved.

Recently, a skin-resident MSC population characterized by expression of ATP-binding cassette subfamily B member 5 (ABCB5) has been found severely reduced in the dermis of diabetic *db/db* mice. This might imply that the function of this cell population is impaired under diabetic conditions, which, as a consequence, might favor poor wound healing in diabetes [50]. Conversely, ABCB5^+^ MSCs were shown to respond to inflammatory milieus through multiple cell contact-dependent and paracrine mechanisms [51–53]. In a chronic wound model mimicking human chronic venous ulcers, ABCB5^+^ MSCs shifted the M1 macrophage prevalence toward an M2 phenotype via secretion of IL-1 receptor antagonist (IL-1RA) and rescued impaired angiogenesis in the wound bed [53]. These effects were associated with an acceleration of wound healing, which was also observed in human chronic venous ulcers treated with ABCB5^+^ MSCs [54, 55]. Very recently, angiogenesis- and healing-promoting efficacy of ABCB5^+^ MSCs was demonstrated also in a mouse diabetic wound model [50].

Dermal ABCB5^+^ MSCs cells can be easily accessed from healthy human donors, expanded to a clinical scale and delivered as a good manufacturing practice (GMP)-conforming advanced-therapy medicinal product (ATMP) of proven purity, safety and tolerability [56, 57]. ABCB5^+^ MSCs display a spindle-like, fibroblastoid cell morphology and express the minimal set of mesenchymal lineage markers CD73, CD90 and CD105, in addition to CD29, CD44, CD49e and CD166, whereas no expression of the endothelial lineage marker CD31, the dendritic cell marker CD34, the pan-hematopoietic lineage marker CD45, the monocyte/macrophage marker CD14 and the B lymphocyte antigen CD20 was detectable by flow cytometry [51, 53]. A consistent and significantly increased potential for adipogenic, osteogenic, and chondrogenic lineage differentiation delineates human ABCB5^+^ MSCs from donor-matched ABCB5^–^ dermal fibroblasts [53]. Aiming at developing ABCB5^+^ MSCs for the treatment of human DFUs, we here characterize their angiogenic potential with respect to angiogenic factor expression at the mRNA and protein level, in vitro endothelial trans-differentiation and tube formation potential, and in vivo perfusion-restoring capacity. Building upon these results in conjunction with the existing evidence on the cells’ anti-inflammatory potential we have established three potency assays in order to deliver human skin-derived ABCB5^+^ MSCs as an ATMP with standardized biological activity. Finally, this product was tested in a clinical trial in patients suffering from non-healing, standard treatment-refractory DFUs.

## Methods

### Expansion and isolation of human ABCB5^+^ MSCs

Human ABCB5^+^ MSCs were derived from skin samples obtained from patients undergoing abdominoplasties or other surgical interventions that provide left-over skin tissue after informed written consent was obtained. Cell production was carried out in an EU-GMP grade A cabinet in a grade B clean room under laminar air flow following a validated GMP-conforming protocol as described previously [56]. In brief, after enzymatic digestion of the skin tissue, cells were centrifuged and expanded as unsegregated culture by serial passaging upon adherence selection in an in-house MSC-favoring medium (Ham’s F-10 supplemented with fetal calf serum, L-glutamine, fibroblast growth factor 2 (FGF-2), HEPES, hydrocortisone, insulin, glucose, and phorbol myristate acetate). ABCB5^+^ cells were isolated by antibody-coupled magnetic bead sorting using a mouse anti-human ABCB5 monoclonal antibody directed against the extracellular loop 3 of the ABCB5 molecule [58] (Maine Biotechnology Services, Portland, Maine; GMP purification: Bibitec, Bielefeld, Germany), cryo-preserved in CryoStor® CS10 freeze medium (BioLife Solution, Bothell, WA) containing 10% dimethyl sulfoxide and stored in the vapor phase of liquid nitrogen.

### Hypoxia studies

#### Induction of cell hypoxia

ABCB5^+^ MSCs (3 × 10^5^) were seeded in 750 µl MSC-favoring medium in a culture dish and placed in a hypoxia chamber, which was flushed for 5 min with nitrogen-enriched gas (1% O_2_, 4% CO_2_, 95% N_2_; Air Liquide, Düsseldorf, Germany) at a rate of 20–25 l/min. During cultivation for up to 48 h, the chamber was flushed again after 1 h and 24 h.

#### HIF-1*α* staining

ABCB5^+^ MSCs were centrifuged (Cytospin™; Thermo Fisher, Dreieich, Germany) onto coverslips, fixed with 4% paraformaldehyde solution, permeabilized with 1% Triton™ X-100 (Sigma-Aldrich, Taufkirchen, Germany) in phosphate-buffered saline, blocked with 0.5% bovine serum albumin in phosphate-buffered saline, and stained for HIF-1*α* (for antibodies see Additional file 1: Table S2). Nuclei were counterstained with 4’,6 diamidino-2 phenylindole (DAPI).

#### Quantitative real-time polymerase chain reaction (qPCR)

Total RNA was isolated using the RNeasy® Micro Kit (Qiagen, Hilden, Germany) and reverse-transcribed into cDNA using the Applied Biosystems™ High-Capacity cDNA Reverse Transcription Kit (Thermo Fisher) and the Applied Biosystems™ SYBR Green Mastermix (Thermo Fisher) in a four-step process run in a Mastercycler® Personal thermocycler (Eppendorf, Hamburg, Germany). PCR reactions were run in triplicate in an Applied Biosystems™ StepOne RealTime™ PCR System (Thermo Fisher). Primer sequences are provided in Additional file 1: Table S3. Actin served as housekeeping gene. Primer quality and integrity of the amplified product was confirmed by melting curve analysis. Identity of the PCR products was confirmed by agarose gel electrophoresis. Relative quantification of transcript levels was determined using the 2^−ΔΔCt^ algorithm.

#### Enzyme-linked immunosorbent assay (ELISA)

VEGF concentration in the cell culture supernatant was measured using the Invitrogen VEGF Human ELISA Kit (Thermo Fisher), according to the manufacturer’s instructions. Assays were run in triplicate.

### Trans-differentiation studies

#### Angiogenic trans-differentiation assay

ABCB5^+^ MSCs (1 × 10^6^) were seeded in 24-well culture plates and cultured for up to 96 h in culture medium supplemented with 200 ng/ml recombinant human (rh) VEGF (Sigma-Aldrich), 1000 ng/ml rhFGF-2 (CellGenix, Freiburg, Germany) and 1000 ng/ml rh platelet-derived growth factor-BB (PDGF-BB; R&D Systems, Wiesbaden, Germany). Trans-differentiation and proliferation activity were assessed by CD31 and Ki67 staining, respectively (for antibodies see Additional file 1: Table S2). Nuclei were counterstained with DAPI. All experiments were performed in triplicates. Human umbilical vein endothelial cells (HUVECs; 5 × 10^5^; Thermo Fisher) served as positive control.

#### Tube formation assay

ABCB5^+^ MSCs (1 × 10^5^/ml or 1.5 × 10^5^/ml) and HUVECs (0.5 × 10^5^/ml or 1 × 10^5^/ml) were seeded on Geltrex™ (Thermo Fisher)-coated culture plates and incubated at 37 °C for 19–22 h (ABCB5^+^ MSCs) and 16–18 h (HUVECs). For examination of cell viability, cells were stained with calcein acetoxymethylester (Thermo Fisher; 1:10,000, 30 min, 37 °C). Tube formation and calcein fluorescence were evaluated microscopically (EVOS™ FLoid™ cell imaging station).

### Animal studies

#### Hindlimb ischemia (HLI) induction and post-surgical care

Male OF1 mice (Charles River Laboratories, Saint-Germain-Nuelles, France) were anesthetized with 2% isoflurane in 100% oxygen, and the inner faces of both hindlimbs were carefully shaved. After local disinfection, an about 1-cm skin incision was made on the left hindlimb from the inguinal region to the bifurcation region of the femoral artery into the saphenous and popliteal artery. The femoral artery and vein were dissected from the nerve. The femoral artery/vein block was ligated proximally by two 8–0 ties placed just distally from the superficial epigastric artery, and distally by two 8–0 ties placed just proximally from the bifurcation of the femoral artery into the saphenous and the popliteal artery. After cutting the femoral artery/vein block between the two proximal and between the two distal ties, the femoral artery/vein block was removed. When necessary, major branches such as the lateral circumflex femoral artery were ligated to avoid bleeding.

Thereafter, subcutaneous tissue and skin were closed with non-resorbable sutures or clamped with titanium micro clips (WDT, Garbsen, Germany). Postoperative care included pain management by injection of buprenorphine (Buprenovet, Bayer; 0.1 mg/kg) once directly after surgery or flunixin meglumine (2.5 mg/kg twice daily) during 3 days and daily local wound care with an antiseptic healing cream (Dermaflon, Pfizer).

#### *Injection of ABCB5*^+^*MSCs*

On the day after surgery, mice were anesthetized with isoflurane to receive intramuscular injections at the ischemic limb of human ABCB5^+^ MSCs suspended in Ringer’s lactate solution containing 2.5% human serum albumin and 0.4% glucose at concentrations between 1 × 10^6^ and 1 × 10^8^ cells/ml, as required. Cell doses, injection volumes and sites are given in the *Results* section.

#### Blood perfusion measurement

Animals were anesthetized with isoflurane and placed on a warming platform in a supine position for imaging at the internal face of the thighs. Hindlimb blood flow was measured before and immediately after surgery (day 1) and on days 3, 5, 7, 14, 21 and 28 by real-time laser Doppler blood perfusion imaging (LDPI; PeriCam PSI, Perimed Instruments). The scanned area covered an ellipse framing internal face of the thigh. Blood perfusion was expressed as the ratio between LDPI values in the left (ischemic) and right (non-ischemic) limb.

#### Histopathology

After sacrifice, the left, ischemic thigh and gastrocnemius muscles were preserved in 10% neutral buffered formalin solution, embedded in paraffin wax, cut to 2–4 µm thickness, stained with hematoxylin and eosin, and inspected by conventional light microscopy. Histopathological findings were quantified according to the scoring system for local cellular and tissue responses described by ISO 10993-6:2007 [59], evaluating the criteria polymorphonuclear cells, lymphocytes, plasma cells, macrophages, giant cells, myofiber degeneration, myofiber regeneration, necrosis, neovascularization, fibrosis, fatty infiltrates and mineralization. Neovascularization was semi-quantitatively quantified as 0 = none, 1 = minimal capillary proliferation, focal, 1–3 buds, 2 = groups of 4–7 capillaries with supporting fibroblastic structures, 3 = broad band, and 4 = extensive band of capillaries with supporting fibroblastic structures.

#### Immunohistochemistry

After sacrifice, left, ischemic thigh muscles were preserved in 10% neutral buffered formalin solution, which was replaced after 24–48 h with 70% ethanol. Immunohistochemical staining for CD31 was performed using rabbit anti-human/mouse CD31 (ab28364, Abcam; dilution 1:50) and dextran polymer-horseradish peroxidase-labeled anti-rabbit IgG (DAKO EnVision® + , K4010, Agilent) for detection. CD31 expression was semi-quantitatively quantified as 0 = none, 1 = minimal, 2 = slight, and 3 = moderate by two independent investigators who were blinded to the treatment.

#### Statistics

One-way ANOVA followed by Dunnett’s test was used to compare LDPI ratios versus baseline and neovascularization and CD 31 expression in the cell-treated groups versus control.

### Clinical trial

#### Patients

Adults (18–85 years) with diabetes mellitus type 2 (hemoglobin A1c < 11%) were eligible if they had a neuropathic diabetic plantar foot ulcer (Wagner grade 1 or 2, 1–50 cm^2^), confirmed by vibration sense testing (128-Hz Rydel-Seiffer tuning fork) without the presence of significant arterial disease (ankle-brachial index ≥ 0.7 or transcutaneous oxygen pressure > 40 mmHg or as per Doppler ultrasonography).

Main exclusion criteria were acute Charcot foot, active osteomyelitis, treatment-requiring ulcer infection, adjacent or chronic skin disorders, skin malignancies, acute or untreated deep vein thrombosis, need for hemodialysis, surgical procedures within 2 months and use of active wound care agents within 2 weeks prior to treatment, and current use of systemic immunosuppressants, cytotoxics or glucocorticoids.

#### Trial design

The study was a national, multicenter (eight sites in Germany), open-label, single-arm, phase I/IIa trial comprising three periods: standard-of-care screening (≥ 6 weeks), treatment and efficacy follow-up (weeks 1–12), and safety follow-up period (until end of month 12). The trial was performed in accordance with the Declaration of Helsinki and local regulations and approved by the ethical committees of all participating study sites. Patients gave written informed consent prior to trial participation.

#### Interventions

Treatment consisted of up to two topical applications of 2 × 10^6^ allogeneic ABCB5^+^ MSCs (suspended in Ringer’s lactate solution containing 2.5% human serum albumin and 0.4% glucose [56]) per cm^2^ wound area on day 0 and at week 6. The cells were manufactured as a GMP-conforming standardized ATMP (for main product release data see Additional file 1: Table S4). Originally, only one cell application was planned. The second application was amended to the protocol only after data from a first-in-human trial on chronic venous ulcers [54] suggested that a second cell dose at 6 weeks after the first cell dose might provide additional benefit for chronic wound healing. Cell application could be preceded by an optional wound debridement at the investigator’s discretion followed by waiting until the bleeding had entirely stopped. For cell application, a suspension containing 1 × 10^7^ ABCB5^+^ MSCs/ml was applied onto the wound surface, delivering 2 × 10^6^ ABCB5^+^ MSCs/cm^2^ wound surface area. Thereafter, the cells were allowed to settle for 15–30 min, optionally fixed in place with fibrin gel (Tisseel®; Baxter, Unterschleißheim, Germany), and then the wound was covered with a waterproof film dressing (Tegaderm™; 3 M, Neuss, Germany). On the following day (≥ 12 h after cell application), the film dressing was replaced by a microbe-binding dressing (Cutimed® Sorbact® tamponade or compress; BSN, Hamburg, Germany), which was changed again 1–2 days later. Additionally, patients received standard care until week 12 including glycemic control, ulcer debridement, appropriate wound dressings (*i.e.,* microbe-binding tamponade of cavities and exudate-absorbent foam dressing for coverage), and antibiotics if required. All patients had to use offloading devices including cast devices or individually fitted therapeutic footwear [19, 60, 61].

#### Outcome measures

Primary efficacy endpoint was percent wound surface area reduction at week 12 or last available post-baseline measurement. Secondary efficacy endpoints were percent and absolute wound surface area reduction at predefined visits, proportion of patients achieving complete and 30% wound closure, time to complete and to 30% wound closure, granulation, epithelialization, wound exudation, time to amputation at the target leg, pain and life quality. Safety outcome measures included adverse events (during the whole study period) and vital signs, changes in physical examination findings and time to amputation of the target leg (during efficacy follow-up).

#### Outcome determination

Wound surface area determination followed a multistep approach combining computerized evaluation (PictZar® planimetry software; BioVisual, Elmwood Park, NJ, USA; 98% accuracy, 94% inter-rater reliability, 98% intra-rater reliability according to a validation and reliability study [62]) of standardized photographs and depth measurements using a wound measuring probe, to account for the typical three-dimensional shape of DFU wounds, *i.e.,* consisting of wound floor, side wall and, occasionally, not visible tunneling or undermining areas. For details of the measuring and calculation algorithm see Additional file 2: Methods S1. Formation of granulation and epithelial tissue was estimated by the investigator in % of wound area from standardized wound photographs. Wound exudation was rated by the investigator as low (dry), moderate (moist), and high (wet) according to the criteria defined by the World Union of Wound Healing Societies [63]. Pain was rated by the patient using a 0–10-point numerical rating scale with 0 = no and 10 = worst imaginable pain. Quality of life was assessed using the participant-reported Short Form (36) Health Survey (SF-36) and Dermatology Life Quality Index (DLQI) questionnaires.

#### Sample size

Enrolment followed a Simon optimal two-stage design with responders defined as patients presenting with at least 30% wound surface area reduction at week 12. The sample size required to achieve 80% power at 5% significance level was calculated using PASS 13 software (NCSS, East Kaysville, UT, USA) to be 37 patients. This enabled the option to terminate the trial if ≤ 6 or ≥ 14 of the first 18 treated patients were responders. As in an interim analysis 12 of 18 patients emerged as responders, recruitment was continued. However, by force of the emerging COVID-19 pandemic, the trial was prematurely completed. At that time, 23 patients had been treated.

#### Statistical analysis

Safety assessments were performed on the safety analysis set, which included all patients who received at least one cell dose. Efficacy assessments were performed on the full analysis set (FAS), which included all patients of the safety analysis set who underwent wound surface area assessments at baseline and at least one post-baseline visit, and on the per-protocol set (PP), which included all patients of the FAS who had no major protocol deviations.

If not otherwise stated, normally (D'Agostino–Pearson normality test) distributed parameters are presented as mean ± standard deviation, and non-normally distributed parameters as median and interquartile range (IQR). Statistical significance of percent wound surface area changes from baseline was tested against the null hypothesis (median change = 0) using a two-sided Wilcoxon signed rank test. Time to complete wound closure, time to 30% wound surface area reduction and time to amputation at the target leg were analyzed using the Kaplan–Meier method.

## Results

### Hypoxia studies

#### *Hypoxia-induced HIF-1α activation in ABCB5*^+^*MSCs*

**Fig. 1:**
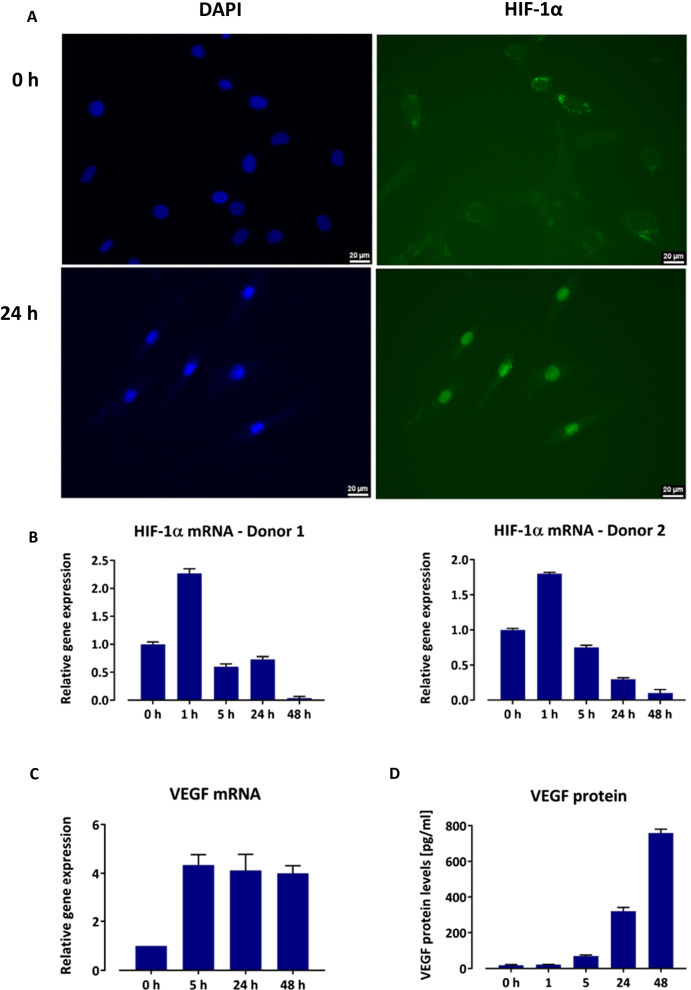
HIF-1*α* and VEGF expression by ABCB5^+^ MSCs during hypoxic culture. **A** Representative immunofluorescence staining of ABCB5^+^ MSCs revealing nuclear translocation of HIF-1*α* at 24 h. Nuclei were counterstained with DAPI. Scale bars: 20 µm. **B** HIF-1*α* mRNA expression by ABCB5^+^ MSCs from two donors, shown as fold expression from baseline (normoxic conditions, 0 h). Data are means + SD of three replicates. **C** VEGF mRNA expression by ABCB5^+^ MSCs, shown as fold expression from baseline (normoxic conditions, 0 h). Data are means + SD of three donors. **D** VEGF protein secretion by ABCB5^+^ MSCS, measured as VEGF protein concentration in culture supernatant. Data are means + SD of three replicates from a representative donor

Prior to hypoxic incubation, HIF-1*α* protein was mainly detectable in the cytoplasm. During hypoxic incubation, cytoplasmic HIF-1*α* fluorescence decreased while nuclear HIF-1*α* fluorescence increased. At 24 h of hypoxia, HIF-1*α* was mainly detectable in the nuclei, indicating that nuclear translocation has occurred (Fig. 1A). In contrast, on the transcriptional level, HIF-1*α* mRNA expression peaked after 1 h of hypoxic culture and decreased thereafter, dropping down to roughly 10% of the baseline value at 48 h (Fig. 1B).

#### *Hypoxia-induced VEGF mRNA expression and protein secretion in ABCB5*^+^*MSCs*

During hypoxia, VEGF mRNA expression increased about fourfold from baseline at 5 h, remaining on that level during 48 h (Fig. 1C). VEGF protein secretion steadily increased during 48 h of hypoxic culture (Fig. 1D).

#### Angiogenic potency assay

The VEGF ELISA after hypoxic culture was used as a surrogate potency assay to predict the pro-angiogenic bioactivity of GMP-compliantly produced ABCB5^+^ MSCs for use in clinical trials. Since VEGF secretion was highest at 48 h of hypoxic culture (Fig. 1D), the 48-h time point was set as temporal endpoint for potency testing. In validation studies (not shown), a VEGF concentration in the supernatant of ≥ 46.9 pg/ml, corresponding to an optical density threshold < 3.0 in the serial standard dilution of the ELISA kit, was validated to reliably enable qualitative detection and, therefore, defined as acceptance criterion for cell batch release. For the potency data of the cell batches used in the present clinical trial see Additional file 1: Table S4.

### Trans-differentiation studies

#### Growth factor-stimulated endothelial trans-differentiation

**Fig. 2:**
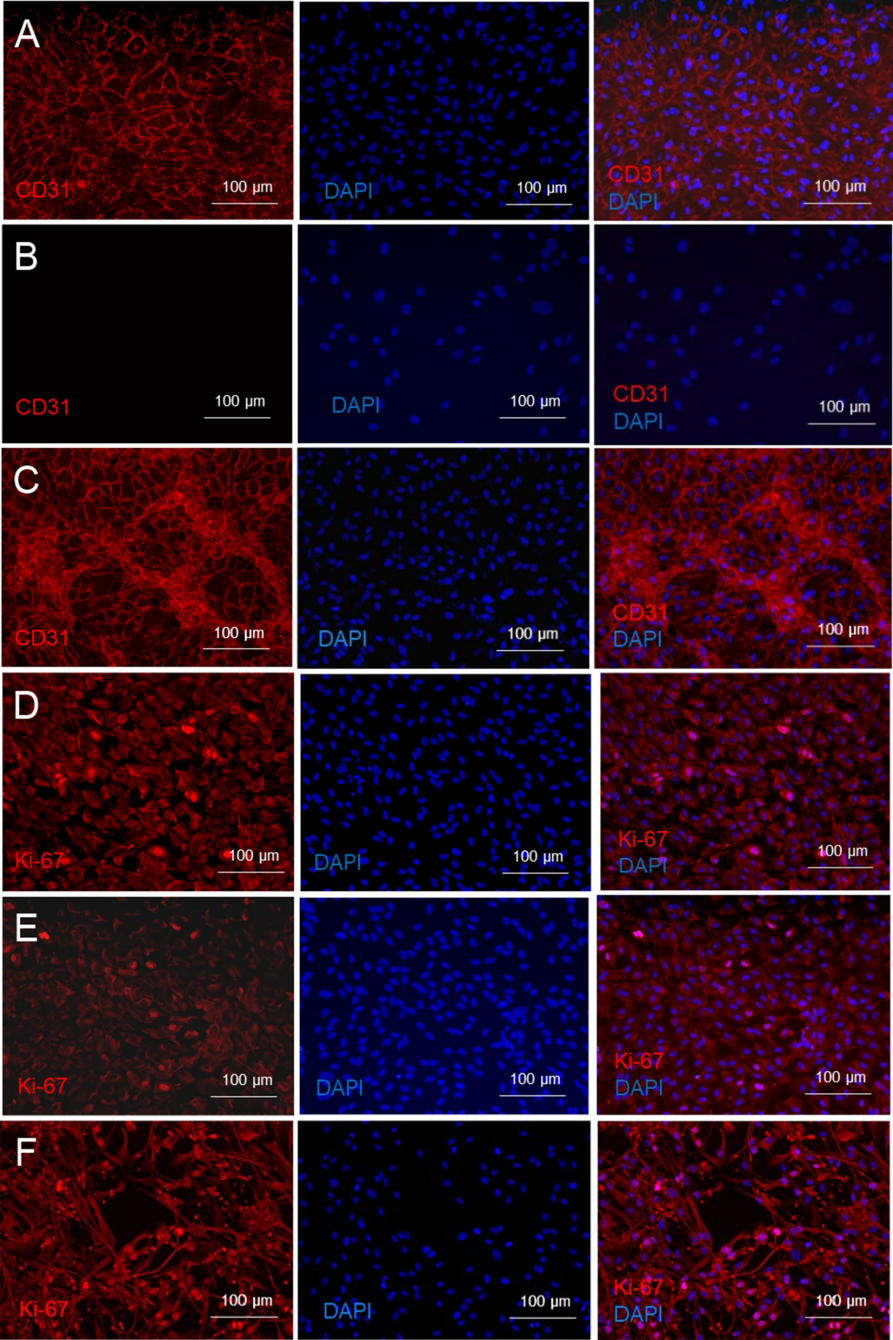
Endothelial trans-differentiation of ABCB5^+^ MSCs. **A** Co-stimulation for 96 h with 200 ng/ml VEGF, 1000 ng/ml FGF-2 and 1000 ng/ml PDGF-BB elicited angiogenic trans-differentiation of ABCB5^+^ MSCs as revealed by CD31-positive (red) staining. **B** ABCB5^+^ MSCs cultured without growth factor supplementation served as negative control. **C** HUVECs served as positive control. **D–F** Proliferative activity of ABCB5^+^ MSCs stimulated to undergo endothelial trans-differentiation. **D** ABCB5^+^ MSCs were stimulated for 96 h with 200 ng/ml VEGF, 1000 ng/ml FGF-2 and 1000 ng/ml PDGF-BB. Proliferative activity was assessed by Ki67 staining (red). **E** ABCB5^+^ MSCs cultured without growth factor supplementation served as negative control. **F** HUVECs served as positive control. Nuclei were counterstained with DAPI (blue). Representative images of three independent experiments

After 96-h culture in medium supplemented with 200 ng/ml VEGF, 1000 ng/ml FGF-2 and 1000 ng/ml PDGF-BB, ABCB5^+^ MSCs underwent endothelial trans-differentiation as revealed by CD31 expression (Fig. 2A–C). Trans-differentiation was accompanied by an enhanced proliferative activity as evidenced by Ki67 staining (Fig. 2D–F).

#### Tube formation on gel matrix

**Fig. 3:**
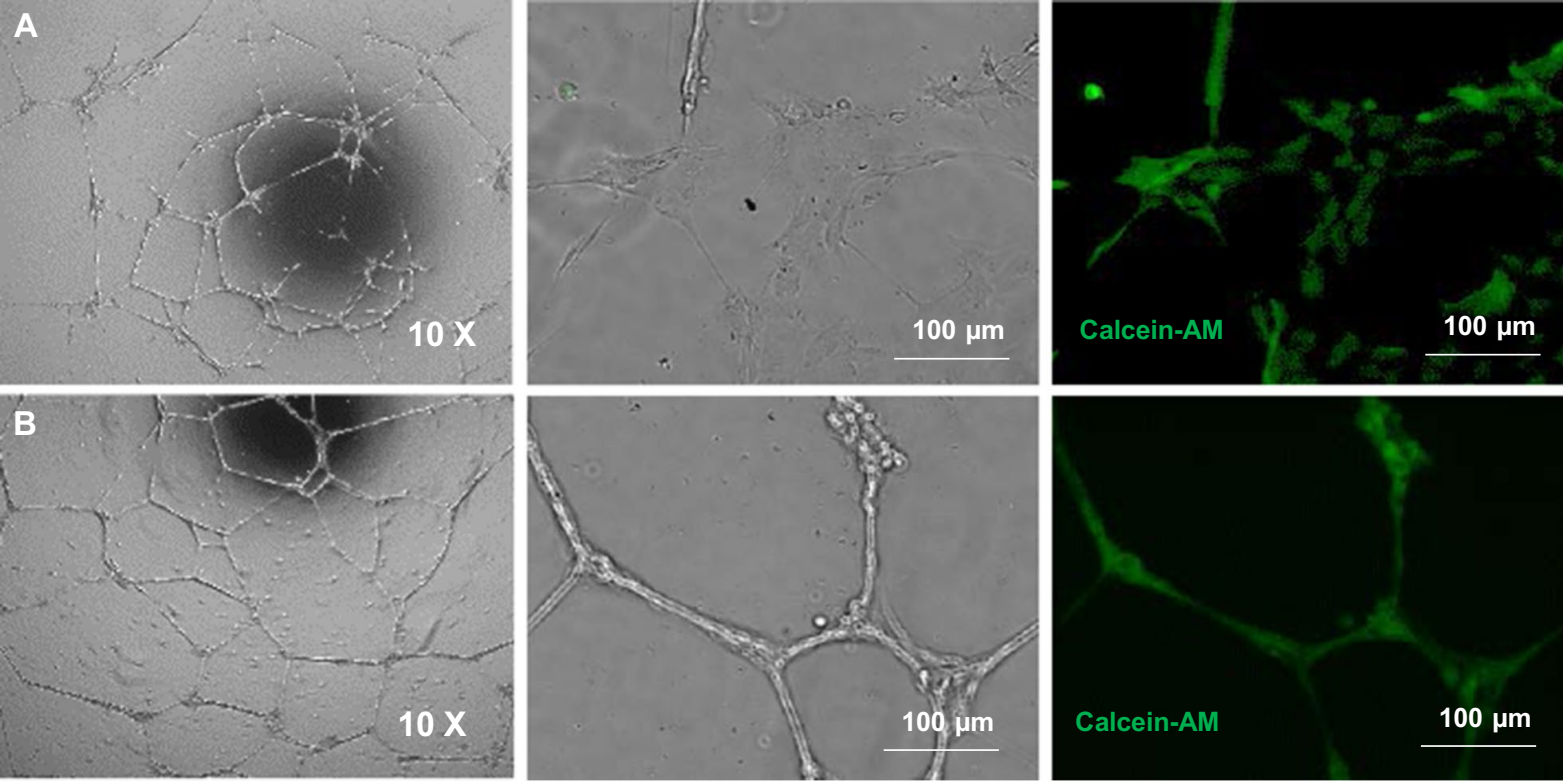
Tube formation assays. **A **Human ABCB5^+^ MSCs and **B** HUVECs were cultured for 18–20 h on Geltrex™ matrix. Calcein staining (green) demonstrates viability (*i.e.,* metabolic activity) of tubular structure-forming cells

After 18–20-h cultivation on Geltrex™ gel matrix, ABCB5^+^ MSCs formed capillary-like structures similar to HUVECs that were used as positive control. The tubular structures stained positive for calcein, demonstrating viability (defined as metabolic activity measured by conversion of calcein acetoxymethylester to calcein) of the tube-forming cells (Fig. 3).

#### Trans-differentiation potency assay

The tube formation assay was used as a surrogate potency assay to predict the trans-differentiation capacity of GMP-compliantly produced ABCB5^+^ MSCs for use in clinical trials. For grading, tube formation of ABCB5^+^ MSCs was semi-quantitatively classified into six categories ranging from 1 = tubular branches of several cells forming a defined network-like structure to 6 = no tubular branches visible (for a more detailed description of all categories see Additional file 1: Table S4, with ≤ 3 in at least one of the two seeded cell concentrations (1 × 10^5^/ml and 1.5 × 10^5^/ml) being considered as successful angiogenic differentiation. For the potency data of the cell batches used in the present clinical trial see Additional file 1: Table S4.

### Animal studies

#### Blood flow recovery in surgically induced HLI

**Fig. 4:**
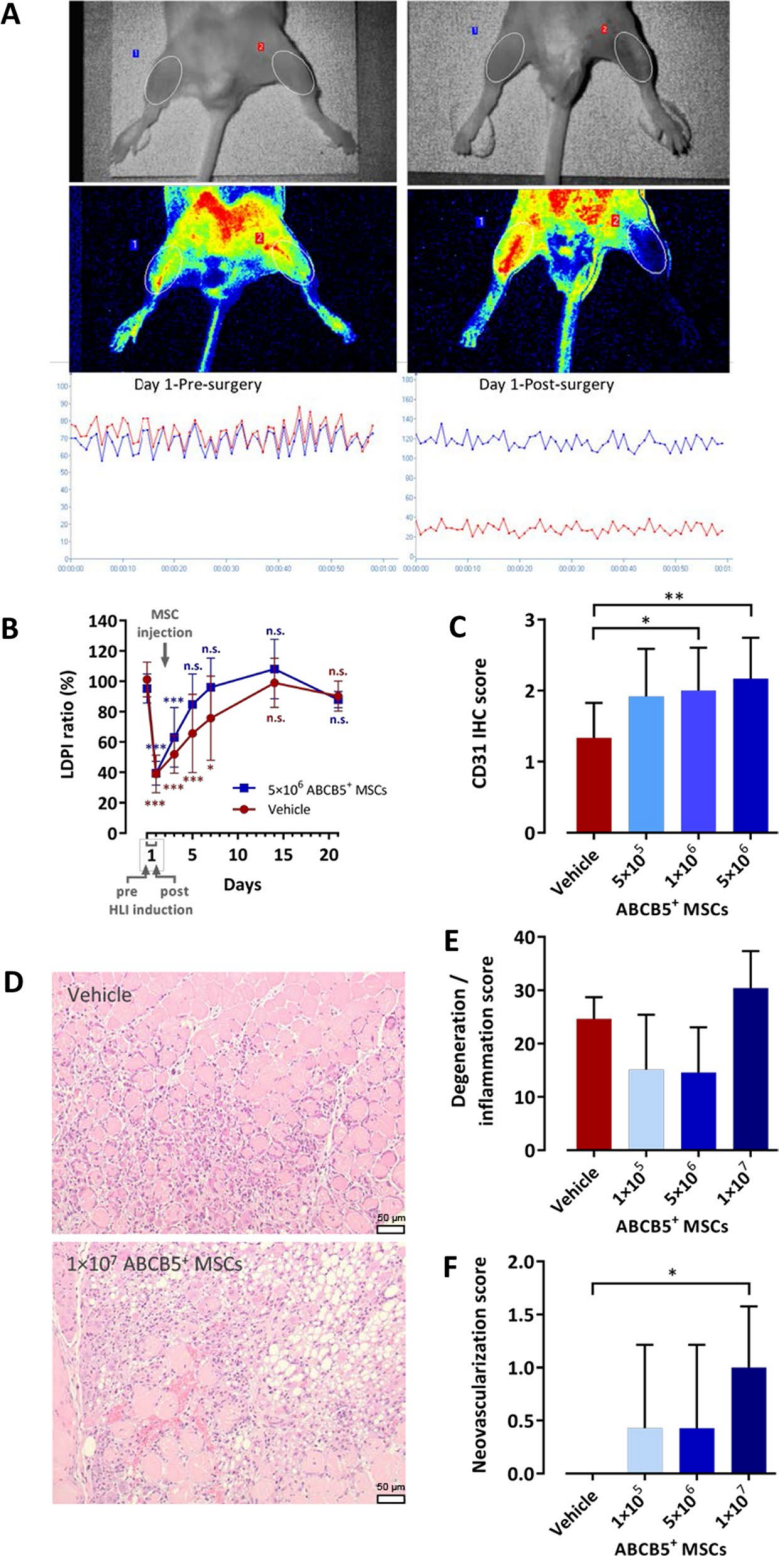
Blood flow recovery and neovascularization following surgically induced HLI in OF1 mice. **A** Representative LDPI acquisition before and immediately after HLI induction, illustrating the experimental setup. Scanned areas are marked by ellipses; the warmest color (intense red) represents 200 perfusion units. Graphs show mean perfusion unit during 1 min in the non-ischemic (blue) and ischemic (red) thigh. **B** LPDI ratio between the ischemic and the non-ischemic limb in mice treated with 5 × 10^6^ ABCB5^+^ MSCs or vehicle. Means with SD of n = 10 (day 1), n = 9 (day 3), n = 8 (day 5; days 7–21 MSCs) and n = 7 (days 7–21 vehicle) animals. **C–F** Immunohistochemical and histopathological evaluation of the ischemic hindlimb muscles at 6 days after HLI induction in mice treated with ABCB5^+^ MSCs or vehicle injected into the ischemic limb 24 h after surgery. **C** CD31 expression in the thigh muscles, presented as mean (SD) IHC score, with 0 = none, 1 = minimal, 2 = slight, and 3 = moderate, of n = 12 animals. **D** Representative H&E sections of the gastrocnemius muscle from a vehicle- and an MSC-treated mouse, showing inflammatory and degenerative lesions in both mice and increased neovascularization in the MSC-treated mouse. Scale bars: 50 µm. **E** Degenerative and inflammatory processes in the gastrocnemius muscle, presented as mean (SD) summary score according to ISO 10993–6:2007 of *n* = 6 (vehicle) and *n* = 7 (MSCs) animals. **F** Neovascularization in the gastrocnemius muscle, presented as mean (SD) score, with 0 = none, 1 = 1–3 focal buds, 2 = groups of 4–7 capillaries with supporting fibroblastic structures, 3 = broad band and 4 = extensive band of capillaries with supporting fibroblastic structures, of *n* = 6 (vehicle) and n = 7 (MSCs) animals. *p < 0.05, **p < 0.01, ***p < 0.001 versus baseline (**B**) or vehicle (**C**, **E**, **F**); one-way ANOVA with Dunnett’s post hoc test

Mice (*n* = 10 per group) received 5 × 10^6^ ABCB5^+^ MSCs/animal or vehicle only by intramuscular injection (200 µl injection volume split over 4 injection sites at the internal face of the thigh) at 24 h after HLI induction. During the study, a certain mortality (day 3, 10%; day 5, 20%; day 7, 25%) was observed, which did not differ between groups. Blood perfusion measured by LDPI (Fig. 4A) and expressed as ratio between LDPI values in the ischemic and the non-ischemic limb (Fig. 4B; Additional file 1: Table S5) significantly decreased immediately after surgery in both treatment groups. During the following days the LDPI ratio gradually recovered, reaching baseline levels on day 5 in the MSC-treated group as compared to day 14 in the vehicle-treated group, with the most pronounced difference in LDPI ratio between groups occurring between days 5 and 7 (Fig. 4B; Additional file 1: Table S5). These results confirmed the observations of a preceding pilot study in OF1 mice with surgically induced HLI showing blood flow recovery within 5 days after injection of ABCB5^+^ MSCs but not of vehicle (Additional file 3: Figure S1).

#### CD31 expression in surgically induced HLI

Mice (n = 10 per group) received 5 × 10^5^, 1 × 10^6^ or 5 × 10^6^ ABCB5^+^ MSCs/animal or vehicle by intramuscular injection (200 µl injection volume split over 4 injection sites at the internal face of the thigh) at 24 h post-HLI induction. At day 6, semiquantitative immunohistochemical evaluation revealed a significant increase in mean CD31 expression in the left thigh muscles of mice treated with the two higher cell doses as compared to the vehicle group (Fig. 4C, Additional file 1: Table S6). In a validation study on OF1 mouse and human skin sections, the CD31 antibody showed cytoplasmic staining of endothelium in both species (Additional file 3: Figure S2). Thus, the staining protocol was suitable to picture the formation of capillaries generated from both mice resident cells and administered human MSCs.

#### Neovascularization in surgically induced HLI

Mice received 1 × 10^5^, 5 × 10^6^ or 1 × 10^7^ ABCB5^+^ MSCs/animal (*n* = 7 per group) or vehicle (*n* = 6) by intramuscular injection (100 µl injection volume split over 5 injection sites at the quadriceps, semitendinosus and gastrocnemius muscles) at 24 h post-HLI induction. At day 6, inflammatory and degenerative myofiber reactions were observed in all groups. The mean summary score was lower in the mice treated with the two lower cell doses and higher in the mice treated with the highest cell dose as compared with the control group; however, the differences were not statistically significant (Fig. 4D, E). In contrast, semiquantitative histological evaluation revealed a significant increase in the mean neovascularization score in the left (ischemic) gastrocnemius muscles of mice treated with the highest cell dose as compared to the vehicle group (Fig. 4D, F).

### Clinical trial

#### Progress of the study

**Fig. 5:**
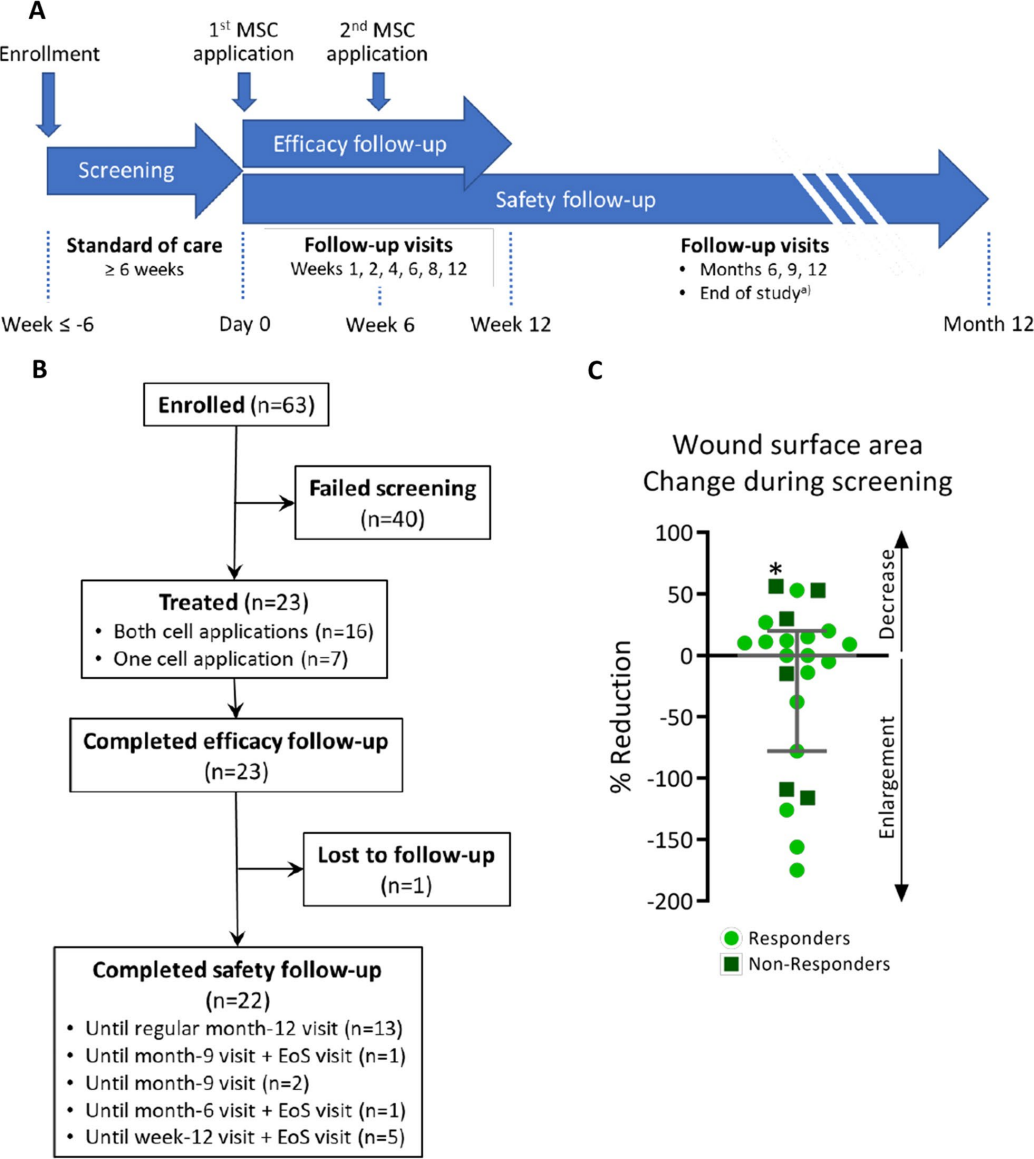
Trial design, study patients and wound surface area during screening. **A** Schematic representation of the trial design. ^a^Only patients who did not reach month-12 visit before 30 June 2020 and were not scheduled for a planned safety follow-up visit in June 2020 were subjected to an end-of-study visit. **B** Study patient flow chart. EoS visit, end-of-study visit [see (a)]. **C** Percent reduction of wound surface area during a ≥ 6-week screening period (median 49 days, range 42–68 days; except for one outlier, whose screening period lasted 118 days, denoted by an asterisk). Error bar represents median and interquartile range

Patients were enrolled between November 2017 and January 2020. Forced by the COVID-19 pandemic, which was associated with critical issues including staffing shortages, impairments of supply chains and increased infection risk for the elderly and/or comorbid study patients, recruitment and treatment were discontinued as of April 2020, and the trial was prematurely completed as of end of June 2020 after consultation with the ethics committee and the regulatory authority. At that time, all treated patients had completed the efficacy follow-up. Patients who had entered the safety follow-up period but were not scheduled for a safety visit in June 2020 were subjected to a supplementary end-of-study visit (Fig. 5A, B).

#### Patients

Totally 63 patients were screened, of which 23 patients (20 men, 3 women) were treated (Fig. 5B). During the screening period, which ranged from 42 to 68 days (one outlier: 118 days; median: 49 days), changes in wound surface area ranged from 56% decrease to 175% enlargement (median change 0%) (Fig. 5C). Baseline characteristics of the treated patients are listed in Table 1.

**Table 1:** Baseline characteristics of all treated patients

Variable		Full analysis set (*N* = 23)
Age, years	Median (range)	62 (49–79)
Sex		
Male	*n* (%)	20 (87)
Female	*n* (%)	3 (13)
Body weight, kg	Median (range)	105 (71–141)
Body mass index, kg/m^2^	Median (range)	33 (26–44)
Target wound surface area, cm^2^	Median (range)	2.6 (1.0–15.2)
Ankle-brachial index	Median (range)	1.1 (0.8–2.0)
Hemoglobin A1c, %	Median (range)	7.2 (5.0–9.8)

Of the 23 treated patients, 7 patients received only one cell application: 3 patients because they had been enrolled under earlier protocol versions before the second application was amended to the protocol, two patients because their wounds were already closed at the week-6 visit, one patient due to a (not treatment-related) foot fracture, and one patient due to COVID-19 pandemic-related treatment discontinuation. One patient was lost to follow-up after the month-9 safety visit (Fig. 1B). Three patients had major protocol deviations: use of prohibited medication (active wound care agent), delayed week-12 visit, improper off-loading. These patients were analyzed in the FAS (*N* = 23) but excluded from the PP (*N* = 20).

#### Efficacy outcomes

**Fig. 6:**
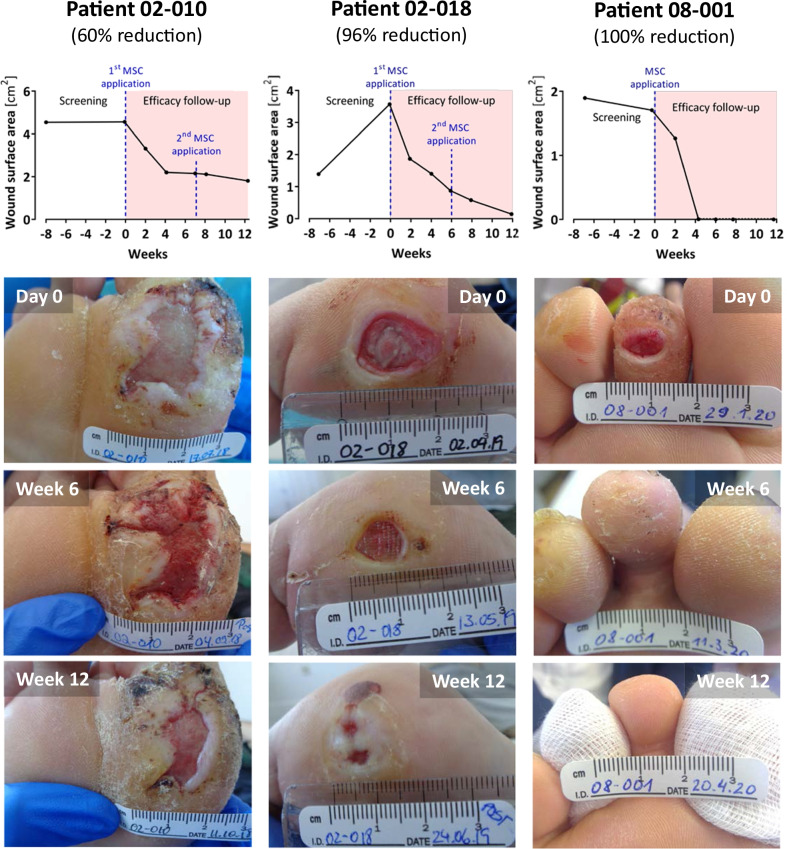
Wound healing progress during the treatment and efficacy follow-up period. Shown are three representative patients in the subgroup of responders. All patients had consented to publication of the photographs

**Fig. 7:**
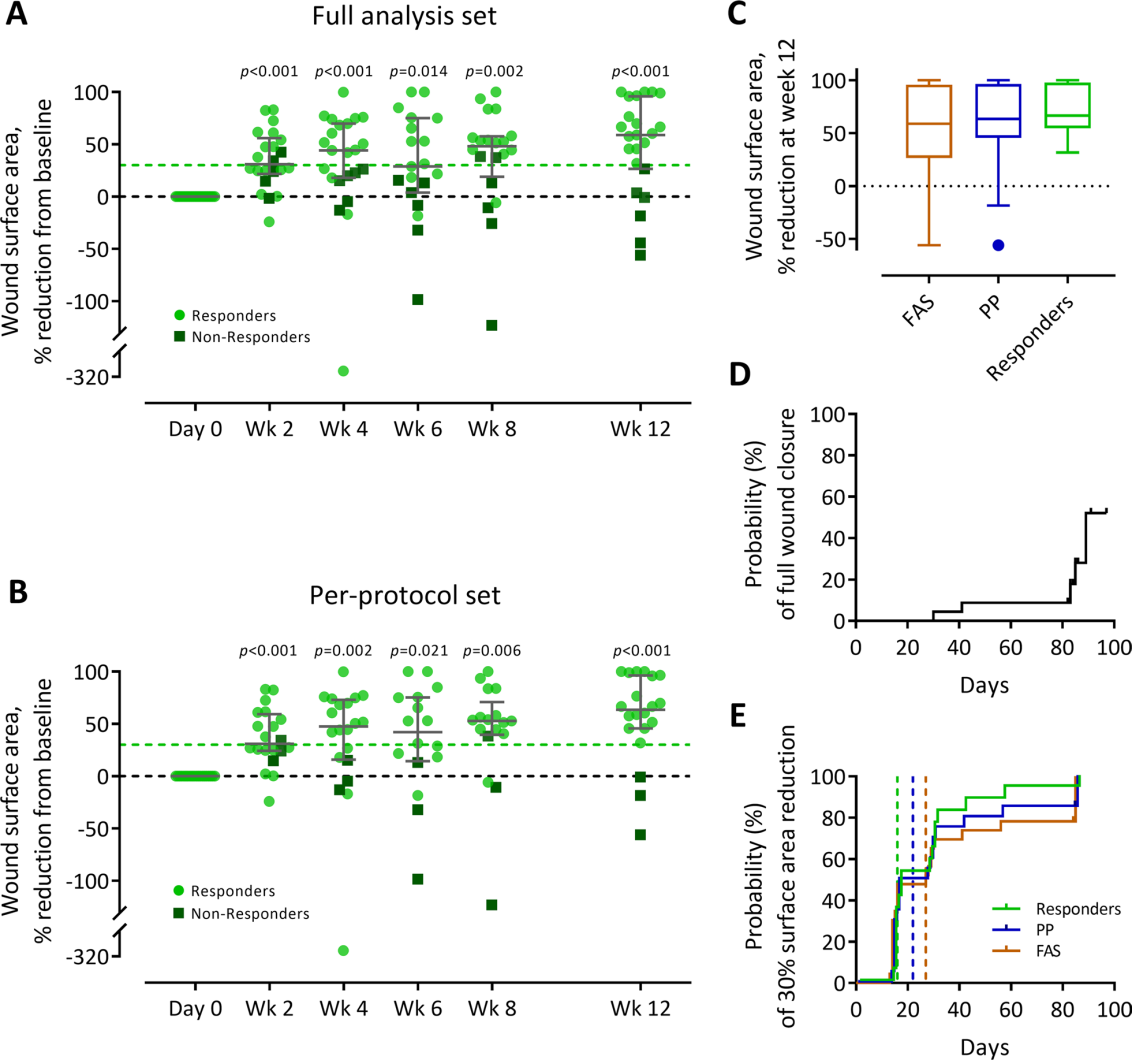
Wound surface area reduction in DFU patients treated with ABCB5^+^ MSCs. **A–B** Percent wound surface area reduction from baseline during the treatment and efficacy follow-up period in the full analysis set (**A**) and per-protocol set (**B**). Patients who presented with wound surface area reductions of at least 30% from baseline (indicated by light green dashed lines) at week 12 were considered responders. Error bars indicate median and interquartile range; *p* values (two-sided Wilcoxon signed rank test) indicate statistical significance of changes from baseline. **C** Tukey’s boxplots of the primary efficacy outcome parameter, % wound surface area reduction from baseline at week 12, in the full analysis set (FAS, *N* = 23), per-protocol set (PP, *N* = 20) and responders (*i.e.,* patients who presented with at least 30% wound surface area at week 12; *N* = 17). **D-E** Kaplan–Meier plots for the time to full wound closure (**D**) and first 30% surface area reduction (**E**) in the FAS, PP and responders. Patients without event were censored at the date of the last available wound surface area assessment (indicated by small vertical ticks). Vertical dashed lines indicate median time to event (not reached for full wound closure)

The wound healing progress of three representative responders is illustrated in Fig. 6. The primary efficacy outcome, median wound surface area reduction from baseline at week 12, was 59% (IQR: 27–96%, FAS) and 64% (IQR: 46–96%, PP) (*p* < 0.001 in both sets) (Fig. 7A–C).

A summary of the secondary efficacy outcomes is given in Table 2.

**Table 2:** Summary of the main secondary efficacy outcomes

Parameter	Full analysis set (*N* = 23)	Per-protocol set (*N* = 20)	Source^a^
Absolute wound surface area reduction			
Change from baseline at week 12 (cm^2^)^b^	1.7 (0.3–2.8)	2.0 (0.9–2.9)	Additional file 1: Table S7
Complete wound closure			
Patients with complete closure at week 12, *n* (%)	6 (26)	6 (30)	Additional file 1: Table S8
Patients with complete closure at any time up to week 12, (%)	6 (26)	6 (30)	Additional file 1: Table S8
Time to complete closure, days^c^	Not reached	Not reached	Figure 7D
≥ 30% wound surface area reduction			
Patients with ≥ 30% reduction at week 12 (“Responders”), *n* (%)	17 (74)	17 (85)	Additional file 1: Table S8
Patients with ≥ 30% reduction at any time up to week 12, *n* (%)	19 (83)	18 (90)	Additional file 1: Table S8
Time to ≥ 30% reduction, days^c^	27 (14; 30)	22 (14; 30)	Figure 7E
Reopening after complete wound closure			
Patients with wounds reopened at week 12, *n* (%)	0 (0)	0 (0)	n.a
Exudation			
Wounds with low exudation, *n* (%)			
Day 0	10 (44)	7 (35)	Additional file 1: Table S9
Week 12	12 (52)	10 (50)	Additional file 1: Table S9
Wounds with moderate exudation, *n* (%)			
Day 0	11 (48)	11 (55)	Additional file 1: Table S9
Week 12	10 (44)	9 (45)	Additional file 1: Table S9
Amputation at target leg			
Patients with amputation, *n* (%)	1 (4)	1 (5)	n.a
Time to amputation, days	42	42	n.a
Pain score^b^			
Day 0	1 (0–3)	n.a	Additional file 1: Table S10
Week 12	1 (0–2)	n.a	Additional file 1: Table S10
Quality of life^d^			
Dermatology Life Quality Index^b^			
Day 0	6 (1–12)	n.a	Additional file 1: Table S11
Week 12	4 (0–10)	n.a	Additional file 1: Table S11

*n.a.* not applicable

^a^Detailed results are given in Additional file 1: Tables S7–S11

^b^Median (interquartile range)

^c^Median (95%-CI)

^d^Due to space limitations, SF-36 subscale scores (which remained virtually unchanged during the efficacy follow-up) are not shown here but given in Additional file 1: Table S11

The median percentage wound surface area reduction was already statistically significant (*p* < 0.001) at 2 weeks and, except for the week-6 assessment (which was, however, missed by 4 patients), increased further over time (Fig. 7A, B)

Absolute wound surface area reduction was most pronounced during the first 2 weeks after the first and second MSC application, *i.e.*, from day 0 till week 2 and from week 6 till week 8, respectively (Additional file 1: Table S7).

Complete wound closure was achieved in 6 patients (26% for FAS and 30% for PP; Additional file 1: Table S8). Since less than half of patients experienced complete wound closure during the efficacy follow up, the median time to complete wound closure could not be determined (Fig. 7D).

Wound surface area reduction by at least 30% at week 12 was observed in 17 patients (74% for FAS and 85% for PP) (Additional file 1: Table S8). These patients were considered responders. The median time to first 30% wound surface area reduction was 27 days (95%-CI: 14; 30; FAS) and 22 days (95%-CI: 14; 30; PP) (Fig. 7E).

Due to the nature of DFU morphology, formation of granulation and epithelial tissue was not reliably evaluable.

Most patients demonstrated low or moderate wound exudation. The proportions of patients with low, moderate or high exudation varied slightly over time with a few more patients having low exudation at week 12 than on day 0 (52% vs. 44% and 50% vs. 35% for FAS and PP, respectively) (Additional file 1: Table S9).

An amputation at the target leg until week 12 was reported in one patient. The reason was a fracture of the little toe, which the investigator judged as unrelated to the cell therapy. Since only one patient experienced an amputation during the efficacy follow up, the median time to amputation could not be calculated.

Median pain score was low during the whole 12-week follow-up period (Additional file 1: Table S10). The SF-36 subscale scores remained virtually unchanged, while the median DLQI slightly improved from 6 (1–12) at day 0 to 4 (0–10) at week 12 (Additional file 1: Table S11).

#### Post hoc analyses

**Fig. 8:**
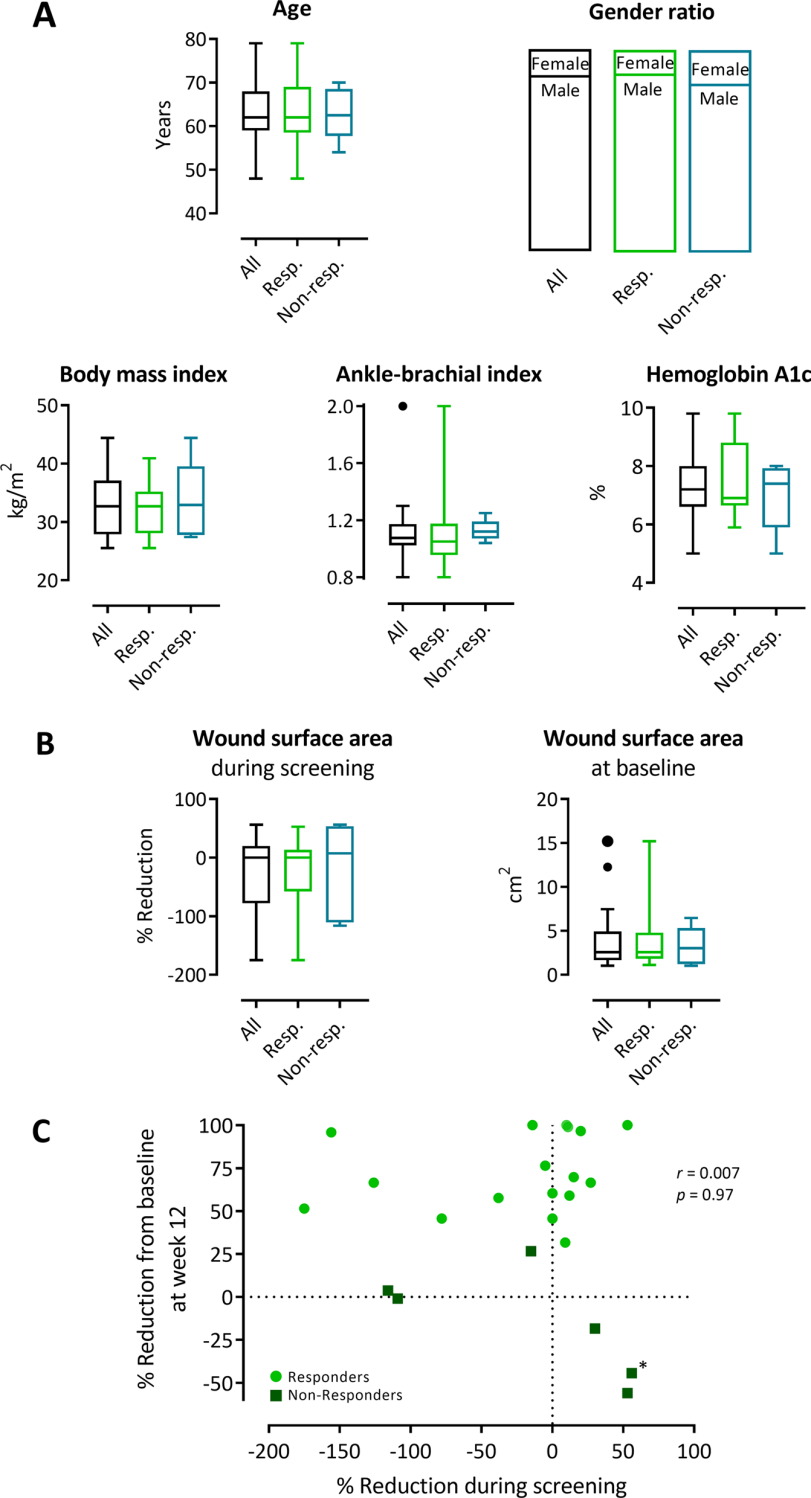
Assessment of potential influences of baseline patient characteristic, baseline wound size and wound surface area reduction during the screening period on response to treatment. Baseline patient characteristics and baseline wound surface area in all treated patients, responders and non-responders. **A–B** Comparisons of baseline patient characteristics (**A**) and of wound surface area reduction during screening and of wound surface area at baseline (**B**) between all treated patients, responders and non-responders. Depicted are Tukey’s boxplots (except for gender ratio); *n* = 23 (all patients; ankle-brachial index: *n* = 22), *n* = 17 (responders; ankle-brachial index: *n* = 16), *n* = 6 (non-responders). Kruskal–Wallis tests followed by Dunn’s multiple comparisons revealed no statistically significant differences between groups (*p* > 0.999 for all comparisons except for ankle-brachial index responders *vs.* non-responders: *p* = 0.697). **C** Spearman’s rank correlation analysis between wound surface area reduction during screening and wound surface area reduction from baseline at week 12. *Asterisk denotes a patient whose screening period lasted 118 days, as compared to 42–68 days (median 49 days) for the other patients

Wound size analyses were additionally performed on the subgroup of responders, *i.e.,* all patients who presented with ≥ 30% wound surface area reductions from baseline at week 12. Baseline patient characteristics, percent change of wound surface area during screening and baseline wound size did not differ between the responders and the non-responders (Fig. 8A, B). All except three responders had achieved first 30% wound surface area reduction within 30 days; median time to first 30% wound surface area reduction was 16 days (Fig. 7E). At week 12, median wound surface area reduction from baseline was 67% (55%–98%) (Fig. 7C), and in 6 of 17 (35%) responders the wound had fully closed.

Since in the trial protocol no threshold values had been specified to exclude patients from study treatment based on their wound surface area changes during the screening period, a Spearman’s rank correlation analysis was performed to test whether there was an association between wound surface area reduction during the screening period and during the efficacy follow-up period. A Spearman’s rank correlation coefficient of 0.007 (95% CI = − 0.429, 0.417; *p* = 0.97) indicated that there was no association between these two parameters (Fig. 8C).

#### Safety outcomes

During the whole study period, 93 treatment-emergent adverse events (TEAEs) were reported in 20 of 23 patients (Table 3). Most TEAEs were mild or moderate; 3 TEAEs reported by two patients were severe. Twelve TEAEs reported by 10 patients were serious (Additional file 1: Table S12). None of the TEAEs was judged related to the cell product.

**Table 3:** Adverse events (SAS)

Event	Number of events	Number (%) of patients
Any adverse event^a^	120	21 (91)
Any TEAE	93	20 (87)
Any serious TEAE	12	10 (43)
Any treatment-related TEAE	0	0 (0)
Frequent TEAEs by MedDRA system organ class^b^		
General disorders and administration site conditions		3 (13)
Edema peripheral		2 (9)
Infections and infestations		15 (65)
Infected skin ulcer		6 (26)
Localized infection		4 (17)
Nasopharyngitis		4 (17)
Wound infection		2 (9)
Injury, poisoning and procedural complications		5 (22)
Ligament sprain		2 (9)
Metabolism and nutrition disorders		4 (17)
Hyperglycemia		2 (9)
Musculoskeletal and connective tissue disorders		4 (17)
Arthralgia		2 (9)
Back pain		2 (9)
Pain in extremity		3 (13)
Skin and subcutaneous tissue disorders		11 (48)
Blisters		6 (26)
Skin ulcer		4 (17)

*MedDRA* Medical Dictionary for Regulatory Activities; *TEAE* Treatment-emergent adverse event; *SAS* Safety analysis set (*N* = 23)

^a^Includes pretreatment-emergent (occurring between giving written consent and first cell application) and treatment-emergent (occurring between first cell application and end of safety follow-up) adverse events

^b^Only TEAEs that were reported by at least 2 patients

During efficacy follow-up, no clinically relevant changes in vital signs occurred (Additional file 1: Table S13). Eighteen physical examination findings that were either not present at baseline or had changed versus baseline were documented in 10 patients (Additional file 1: Table S14). Of these, 10 findings (56%) represented improvements.

## Discussion

Despite well-established strategies for DFU management, treatment is often challenging, and many patients respond only poorly to standard treatment. Experimental approaches that directly target defective pathways in the wound tissue, including blockade of IL-1β by a neutralizing antibody or IL-1RA [30, 64, 65], stabilization of HIF-1*α* using prolyl hydroxylase inhibitors, iron chelator or protein–protein interaction inhibitors [35, 37, 66, 67] or topical supplementation of pro-angiogenic growth factors [39, 68], have accelerated wound healing in diabetic mice. However, the clinical translation of these approaches has been hampered by various hurdles including safety concerns, short half-lives and/or the requirement of specific delivery systems [67, 69, 70]. In contrast to single-drug approaches, therapeutically applied MSCs are considered, by sensing their environment for hypoxia or other stress signals and making use of multiple pathways, to respond more accurately according to the physiological needs [43, 44].

Here, we demonstrate that skin-derived ABCB5^+^ MSCs adaptively activate the proangiogenic HIF-1 pathway in response to hypoxic conditions. Activation occurred through posttranslational stabilization of HIF-1*α* protein (which at normoxia is subject to rapid degradation), which resulted in cellular accumulation and translocation to the nucleus (Fig. 1A). HIF-1*α* pathway response to hypoxia became also evident at the transcriptional level, albeit with different kinetics: Whereas HIF-1*α* protein accumulation and nuclear translocation was most pronounced at 24 h, HIF-1*α* mRNA levels, after initial upregulation to peak levels already at 1 h, progressively decreased during sustained hypoxia, having returned to baseline levels at 5 h and dropping further thereafter (Fig. 1B). Similar kinetics of HIF-1*α* mRNA expression in response to hypoxia have been observed in various cell types including endothelial cells and have been ascribed to a negative feedback loop that counteracts excessive HIF-1*α* protein accumulation during prolonged hypoxic conditions [71]. Interestingly, in mice transplanted with subcutaneous gel grafts cellularized with MSCs and endothelial progenitor cells (EPCs), genetic ablation or pharmacologic inhibition of HIF-1*α* in the MSCs completely abrogated experimental vessel formation in the gel graft, while HIF-1*α* deletion in the EPCs had no effect on vasculogenesis [72]. Thus, even though HIF-1*α* pathway activation is basically a common mechanism by which a cell adapts to reduced oxygen tension, HIF-1*α* stabilization in MSCs was considered a crucial event in cell-based therapeutic vasculogenesis [72]. In ABCB5^+^ MSCs, HIF-1 pathway activation was accompanied by about fourfold upregulation of VEGF transcription (Fig. 1C), which eventually resulted in a substantial increase in VEGF protein secretion (Fig. 1D).

In addition to paracrine VEGF secretion under hypoxic conditions, ABCB5^+^ MSCs proved capable of adopting phenotypic and functional characteristics of endothelial cells in vitro, as demonstrated by expression of CD31 when cultured in growth factor-supplemented medium (Fig. 2A) and formation of capillary-like structures similar to HUVECs when seeded on gel matrix (Fig. 3). This suggests that ABCB5^+^ MSCs can trans-differentiate into endothelial-lineage cells, and might imply that, beyond serving paracrine proangiogenic functions to promote vascular regeneration, ABCB5^+^ MSCs could even directly participate in neoangiogenesis in the injured tissue. Previously, ABCB5^+^ MSCs have shown superior homing and engraftment to mouse skin wounds as compared to bone marrow-derived MSCs [73]. Graft survival was demonstrated in the skin against a fully allogeneic barrier (BALB/c ABCB5^+^ MSCs administered to C57/BL6 mice) for at least 17 days [51]. Moreover, in an NSG mouse wound model, significant detection of human-specific CD31 DNA in the wound tissue at 13 days after topical application of human ABCB5^+^ MSCs has indicated that endothelial trans-differentiation of these cells can actually occur in vivo [54]. Still, whether therapeutically applied ABCB5^+^ MSCs indeed become integrated in the regenerating vasculature remains to be elucidated.

To investigate the vascular regenerative potential of ABCB5^+^ MSCs in vivo, we studied the effects of intramuscular cell injection on perfusion restoration in a mouse HLI model. As required from a drug regulatory perspective [74, 75], we did not use mouse ABCB5^+^ MSCs but tested the ATMP made of human ABCB5^+^ MSCs. Importantly in this regard, a significant body of studies comparing regenerative and anti-inflammatory effects of rodent and human MSCs in immunocompetent rodent models have confirmed a comparable efficacy of allogeneic and xenogeneic MSCs [76–78]. This has been attributed to the low levels of HLA and co-stimulatory molecules expressed by MSCs and by the fact that both, allogeneic and xenogeneic MSCs, survive only for a limited time in the host, before species differences could manifest [76]. In the HLI model, ABCB5^+^ MSCs markedly accelerated perfusion recovery as measured by LDPI (Fig. 4B; Additional file 1: Table S5). At the microscopic level, ABCB5^+^ MSCs significantly increased the vascularization assessed by CD31 immunostaining (Fig. 4C; Additional file 1: Table S6) and enhanced the proliferation of capillaries in the ischemic muscles (Fig. 4D,F).

Beside proangiogenic capacity based on paracrine activity and endothelial differentiation, a third principal mode of action by which MSCs perform their effects over injured tissue is through interaction with the immune system. While immune modulation has not been addressed in the present study, previous studies have demonstrated that ABCB5^+^ MSCs respond to inflammatory milieus through multiple cell contact-dependent and paracrine mechanisms [51–53], including dampening the IL-1β-driven inflammation in chronic wounds by adaptive IL-1RA release, thereby shifting the prevalence of M1 toward proangiogenic and repair-promoting M2 macrophages in the wound tissue [53].

With respect to these three principal modes of actions we have developed and established three potency assays to guarantee biological functionality and predict clinical effectiveness of therapeutically applied ABCB5^+^ MSCs: (i) VEGF secretion under hypoxic conditions to evaluate the angiogenic potency, (ii) tube formation on extracellular matrix gel to evaluate the endothelial trans-differentiation capacity, and (iii) IL-1RA secretion after cocultivation with M1-polarized macrophages to evaluate the immunomodulatory potency (Additional file 1: Table S4).

Based on the *in vitro* and preclinical observations, we investigated ABCB5^+^ MSCs as a potential option for adjunctive treatment of DFUs. In the patient population studied, the greatest treatment success achieved over the ≥ 6-week screening period (median 7 weeks) with standard care alone was approximately 50% wound surface area reduction in 3 of 23 patients (of whom 1 patient was even treated 118 days), while in about half of the patients the ulcer enlarged (up to 175%) (Fig. 5C). Thus, in line with current DFU treatment guidelines, which recommend to consider adjunctive therapy options for DFUs that did not achieve a 50% area reduction within 4 weeks [19–21] or failed to heal after 4–6 weeks [22] of standard treatment, these ulcers had appeared refractory to standard treatment, indicating an urgent need for an advanced wound closure strategy [19–22, 79].

In this hard-to-heal population, adjunctive topical application of ABCB5^+^ MSCs elicited statistically significant median wound surface area reductions from baseline of 59% (FAS), 64% (PP) and 67% (subgroup of responders) after 12 weeks. Acceleration of wound healing started early, becoming statistically significant (*p* < 0.001) already at 2 weeks (median wound surface area reduction 31% for FAS and PP) (Fig. 7A, B), which indicates that as early as at 2 weeks about half of the patients (48% of FAS and 56% of PP) had passed the predefined threshold value of 30% wound surface area reduction that was considered to classify them as responders (Fig. 7D). At 4 weeks, the median wound surface area reduction was 44% (FAS) and 48% (PP), and 1 patient (4% of FAS and 5% of PP) presented already with full wound closure (Fig. 7A, B), which together revealed that the overall situation had clearly improved as compared to the standard-of-care screening period. Finally, at week 12, 6 of these urgent-need patients (26%, 30% and 35% of FAS, PP and responders, respectively) had reached full wound closure, and it seems reasonable to expect that this rate would increase further if the follow-up period was extended, as suggested by the observation that the median wound surface area reduction was still increasing (Fig. 7A, B).

A variety of other cell-based treatment strategies have been tested in controlled clinical trials for adjunctive treatment of DFUs, including autologous and allogeneic MSCs derived from bone marrow and adipose tissue [80–82], autologous adipose tissue and adipose-derived stromal vascular fraction cells [83, 84], autologous platelet-rich plasma [83, 85–90], allogeneic platelets [91], autologous skin cells [92], skin allografts [93], and various cell-containing skin substitute products [94–99] (for a summary see Additional file 1: Table S15). The reported effects show great variations, ranging from wound surface area reductions and full wound closure ratios within 12–26 weeks that did not significantly differ from the control groups [82, 83, 85, 92] to impressive significant wound surface area reductions from baseline of up to 98% within 80 days [90] and wound closure ratios of up to 100% within 8 weeks [84]. However, in the trials that observed superiority of the investigational treatment over standard treatment, the reported outcomes in the standard treatment (control) groups ranged up to 88% wound surface area reduction [90] and 78% full wound closure ratio [88], indicating that considerable proportions of the treated wounds have not been refractory to standard treatment (Additional file 1: Table S15). In contrast, in the present trial we have focused on a standard treatment-refractory, extremely hard-to-heal population, which needs to be taken into account when comparing the outcomes in the present trial with those of other adjunctive treatment strategies.

In view of the high personal and socioeconomic disease burden of DFUs it has become desirable to identify the patients who are likely to benefit from an adjunctive treatment strategy as early as possible. Basically, the phenomenon that a certain proportion of patients do not respond to the treatment, is widely known across the various MSC therapy approaches in a broad range of diseases, with non-responders potentially amounting up to 60% of the treated patients [100]. A major part of variability in clinical outcomes of MSC therapies has been ascribed to heterogenous products with insufficiently characterized therapeutic potency [49]. In contrast, in the present study, the strongly standardized quality and potency of the cell product (Additional file 1: Table S4) rules out potential differences in quality and bioactivity as a cause of variation in the treatment responses, which is supported by the comparably low non-responder rates of 26% (FAS) and 15% (PP). Importantly, there was no association between the treatment responses and the potency assay data of the applied cells (Additional file 1: Table S4), which indicates that the specified threshold acceptance values for product release are strong enough to guarantee proper biological activity. When comparing potential patient-related negative predictors for DFU healing such as greater wound surface area [101–106] and patient characteristics including older patient age [106–108], male gender [104, 109], very high [110] or very low [108] body mass index, lower ankle-brachial index [109] and lower hemoglobin A1c [111], there were no significant differences between the responders and the non-responders that seem to have contributed to failure of treatment response (Fig. 8A). Clearly, however, the etiology of impaired DFU healing is far more multifactorial, involving, e.g., previous diabetes control, comorbidities, and psycho-social factors [79]. On a cellular level, differential regulation or variations of genes involved in skin barrier function, inflammation or vascularization and blood flow have been associated with impaired DFU healing [112–115]. To further investigate what segregates responders to ABCB5^+^ MSC treatment from non-responders could aid identifying predictors of response which might help distinguishing already before treatment initiation the patients that will likely respond to ABCB5^+^ MSC therapy from those who will not.

Naturally, the conclusions drawn from the present trial are limited by factors typically associated with early-phase trials, particularly a small patient number and an open, non-randomized design. Even though all ulcers had emerged refractory to standard treatment, we cannot rule out that part of the observed improvements has occurred through additional attention and care during the trial. Not least, as discussed above, wound healing can be influenced by various patient-specific factors that were not controlled for.

Despite these limitations, we conclude that the present results support GMP-manufactured dermal ABCB5^+^ MSCs as a potential developable candidate for adjunctive therapy of standard treatment-refractory DFUs, even though in the present trial the majority of responders achieved only partial wound closure. Clearly, partial wound closure is a clinically less meaningful outcome than full wound closure; however, it is considered valid to “indicate relevant biological activity and help guide subsequent trials design” [75]. Importantly, the absence of any treatment-related adverse event during the trial confirmed good tolerability and overall safety of the cell product.

## Conclusions

The present studies demonstrate the in vitro and in vivo angiogenic potential of ABCB5^+^ MSCs based on both, paracrine pro-angiogenic factor secretion and trans-differentiation into endothelial-lineage cells. Together with the wound surface area reduction observed upon topical administration onto chronic DFUs, the results support GMP-manufactured ABCB5^+^ MSCs as a safe, viable candidate for adjunctive therapy of treatment-refractory DFUs and warrant further investigation in a larger randomized controlled trial with a dose-ranging design, an extended efficacy follow-up period and advanced outcome measures in order to validate the benefit and optimize the dose regime.

## Supplementary Information

**Additional file 1**.** Table S1**. Reported healing failure rates of diabetic foot ulcers.** Table S2**. Antibodies used for immunofluorescence evaluation.** Table S3**. Primers.** Table S4**. ABCB5^+^ MSC product release data.** Table S5**. LDPI measurements.** Table S6**. CD31 expression in the ischemic thigh muscle of OF1 mice with surgically induced hindlimb ischemia treated with vehicle or ABCB5^+^ MSCs.** Table S7**. Absolute wound surface area reduction from baseline by visit.** Table S8**. Patients with complete wound closure and with ≥30% wound surface area reduction by visit.** Table S9**. Wound exudation by visit.** Table S10**. Pain score by visit.** Table S11**. Quality of life scores by visit.** Table S12**. Serious treatment-emergent adverse events.** Table S13**. Vital signs.** Table S14**. Changes in physical examination findings from screening visit.** Table S15**. Controlled clinical trials evaluating the efficacy of cell-based adjunctive strategies to treat diabetic foot ulcers.

**Additional file 2. Methods S1**. Determination of the wound surface area.

**Additional file 3. Figure S1**. Validation of the rabbit anti-human/mouse CD31 antibody.** Figure S2**. Blood flow recovery following surgically indcued hindlimb ischemia in OF1 mice.

## Data Availability

The datasets generated and/or analyzed during the current study are available from the corresponding author on reasonable request. For requests regarding ABCB5 monoclonal antibody availability contact Markus H. Frank, Markus.Frank@childrens.harvard.edu.

## Abbreviations

ABCB5: ATP-binding cassette transporter, subfamily B, member 5
ATMP: Advanced-therapy medicinal product
DAPI: 4′,6 Diamidino-2 phenylindole
DFU: Diabetic foot ulcer
DLQI: Dermatology life quality index
ELISA: Enzyme-linked immunosorbent assay
FAS: Full analysis set
FGF-2: Fibroblast growth factor 2
GMP: Good manufacturing practice
HIF**-1**: Hypoxia-inducible transcription factor 1
HLI: Hindlimb ischemia
HUVEC: Human umbilical vein endothelial cells
IL-1β: Interleukin 1β
IL-1RA: IL-1 receptor antagonist
IQR: Interquartile range
LDPI: Laser Doppler blood perfusion imaging
MSC: Mesenchymal stem cell
PDGF-BB: Platelet-derived growth factor-BB
PP: Per-protocol set
qPCR: Quantitative real-time polymerase chain reaction
rh: Recombinant human
SF-36: Short form (36) health survey
TEAE: Treatment-emergent adverse event
VEGF: Vascular endothelial growth factor
