# Supplementary material for: Translational development of ABCB5+ dermal mesenchymal stem cells for therapeutic induction of angiogenesis in non-healing diabetic foot ulcers
Source: Stem Cell Res Ther. 2022 Sep 5;13:455. doi: 10.1186/s13287-022-03156-9 (PMC9444095; doi:10.1186/s13287-022-03156-9)
Supplement: Supplementary file 1 — Additional file 1. Table S1. Reported healing failure rates of diabetic foot ulcers. Table S2. Antibodies used for immunofluorescence evaluation. Table S3. Primers. Table S4. ABCB5+ MSC product release data. Table S5. LDPI measurements. Table S6. CD31 expression in the ischemic thigh muscle of OF1 mice with surgically induced hindlimb ischemia treated with vehicle or ABCB5+ MSCs. Table S7. Absolute wound surface area reduction from baseline by visit. Table S8. Patients with complete wound closure and with ≥30% wound surface area reduction by visit. Table S9. Wound exudation by visit. Table S10. Pain score by visit. Table S11. Quality of life scores by visit. Table S12. Serious treatment-emergent adverse events. Table S13. Vital signs. Table S14. Changes in physical examination findings from screening visit. Table S15. Controlled clinical trials evaluating the efficacy of cell-based adjunctive strategies to treat diabetic foot ulcers. [file 13287_2022_3156_MOESM1_ESM.docx]

**Table S1** Reported healing failure rates of diabetic foot ulcers

| **Ulcer etiology / Clinical characteristics** | **Study type** | **N** | **Percentage of ulcers not healed at** | | | | | | | | **Ref.** |
| --- | --- | --- | --- | --- | --- | --- | --- | --- | --- | --- | --- |
|  |  |  | **4 weeks** | **6 weeks** | **8 weeks** | **12 weeks** | **16 weeks** | **20 weeks** | **12 months** | **6.5 years** |  |
| All / not specified | Prospective, observational | 65 | 79% |  | 57% | 43% |  |  |  |  | [1] |
|  | Control groups of 26 RCTs | 1213 |  |  |  | 62% |  |  |  |  | [2] |
|  | Prospective cohort study | 1088 |  |  |  |  |  |  | 23% |  | [3] |
|  | Prospective cohort study | 704 |  |  |  | 65% | 59% |  | 27% |  | [4] |
|  | US Wound Registry data | 62,964 |  |  |  | 70% |  |  |  |  | [2] |
|  | Retrospective cohort study | 343 |  |  |  |  |  |  | 27% |  | [5] |
|  | Prospective, observational | 347 |  |  |  |  |  |  | 33% |  | [6] |
|  | Prospective, observational | 258 |  |  |  |  |  |  | 35% |  | [7] |
|  | Retrospective cohort study | 108 |  |  |  | 52% |  | 44% | 37% |  | [8] |
|  | Prospective, observational | 89 |  |  |  |  |  |  |  | 23% | [9] |
| Neuropathic | Prospective, observational | 402 |  |  |  | 41% |  | 30% | 13% |  | [10] |
|  | Prospective cohort study | 30 |  |  |  | 47% |  |  |  |  | [11] |
|  | Retrospective cohort study | 72,525 |  |  |  |  |  | 50% |  |  | [12] |
|  | Retrospective cohort study | 27,630 |  |  |  |  |  | 53% |  |  | [13] |
|  | Control groups of five RCTs | 586 |  |  |  | 76% |  | 67% |  |  | [14] |
|  | Retrospective cohort study | 130 |  |  |  |  |  |  | 15% |  | [5] |
|  | Prospective cohort study | 558 |  |  |  |  |  |  | 16% |  | [3] |
| Ischemic and neuro‑ischemic | Prospective cohort study | 505 |  |  |  |  |  |  | 31% |  | [3] |
|  | Retrospective cohort study | 213 |  |  |  |  |  |  | 34% |  | [5] |
| Clinically infected | Prospective, observational | 299 |  |  |  |  |  |  | 55% |  | [15] |
| “Hard-to-heal” (treatment-refractory) | Control group of an RCT | 20 |  | 95% |  | 80% |  |  |  |  | [16] |
|  | Retrospective | 651 |  |  |  |  |  |  | 67-70% |  | [17] |

RCT, Randomized controlled trial

**References**

1. Seth A, Attri AK, Kataria H, Kochhar S, Seth SA, Gautam N. Clinical profile and outcome in patients of diabetic foot infection. Int J Appl Basic Med Res 2019;9:14-19.
2. Fife CE, Eckert KA, Carter MJ. Publicly reported wound healing rates: The fantasy and the reality. Adv Wound Care 2018;7:77-94.
3. Prompers L, Schaper N, Apelqvist J, Edmonds M, Jude E, Mauricio D, et al. Prediction of outcome in individuals with diabetic foot ulcers: focus on the differences between individuals with and without peripheral arterial disease. The EURODIALE study. Diabetologia 2008;51:747-755.
4. Coerper S, Beckert S, Küper MA, Jekov M, Königsrainer A. Fifty percent area reduction after 4 weeks of treatment is a reliable indicator for healing – analysis of a single-center cohort of 704 diabetic patients. Journal Diabetes Complications 2009;23:49-53.
5. Thewjitcharoen Y, Sripatpong J, Krittiyawong S, Porramatikul S, Srikummoon T, Mahaudomporn S, et al. Changing the patterns of hospitalized diabetic foot ulcer (DFU) over a 5‑year period in a multi-disciplinary setting in Thailand. BMC Endocr Disord 2020;20:89.
6. Ha Van G, Amouyal C, Bourron O, Aubert C, Carlier A, Mosbah H, et al. Diabetic foot ulcer management in a multidisciplinary foot centre: one-year healing, amputation and mortality rate. J Wound Care 2020;29:464-471.
7. Xiang J, Wang S, He Y, Xu L, Zhang S, Tang Z. Reasonable glycemic control would help wound healing during the treatment of diabetic foot ulcers. Diabetes Ther 2019;10:95-105.
8. Roth-Albin I, Mai SHC, Ahmed Z, Cheng J, Choong K, Mayer PV. Outcomes following advanced wound care for diabetic foot ulcers: a Canadian study. Can J Diabetes 2017;41:26-32.
9. Ghanassia E, Villon L, Thuan Dit Dieudonné JF, Boegner C, Avignon A, Sultan A. Long-term outcome and disability of diabetic patients hospitalized for diabetic foot ulcers: a 6.5-year follow-up study. Diabetes Care 2008;31:1288-1292.
10. Ince P, Game FL, Jeffcoate WJ. Rate of healing of neuropathic ulcers of the foot in diabetes and its relationship to ulcer duration and ulcer area. Diabetes Care 2007;30:660-663.
11. Dinh T, Tecilazich F, Kafanas A, Doupis J, Gnardellis C, Leal E, et al. Mechanisms involved in the development and healing of diabetic foot ulceration. Diabetes 2012;61:2937-2947.
12. Margolis DJ, Allen-Taylor L, Hoffstad O, Berlin JA. Diabetic neuropathic foot ulcers: the association of wound size, wound duration, and wound grade on healing. Diabetes Care 2002;25:1835-1839.
13. Margolis DJ, Allen-Taylor L, Hoffstad O, Berlin JA. Diabetic neuropathic foot ulcers: predicting which ones will not heal. Am J Med 2003;115:627-631.
14. Margolis DJ, Kantor J, Santanna J, Strom BL, Berlin JA. Risk factors for delayed healing of neuropathic diabetic foot ulcers: a pooled analysis. Arch Dermatol 2000;136:1531-1535.
15. Ndosi M, Wright-Hughes A, Brown S, Backhouse M, Lipsky BA, Bhogal M, et al. Prognosis of the infected diabetic foot ulcer: a 12‑month prospective observational study. Diabet Med 2018;35:78-88.
16. Zelen CM, Orgill DP, Serena T, Galiano R, Carter MJ, DiDomenico LA, et al. A prospective, randomised, controlled, multicentre clinical trial examining healing rates, safety and cost to closure of an acellular reticular allogenic human dermis versus standard of care in the treatment of chronic diabetic foot ulcers. Int Wound J 2017;14:307-315.
17. Sørensen MLB, Jansen RB, Wilbek Fabricius T, Jørgensen B, Svendsen OL. Healing of diabetic foot ulcers in patients treated at the Copenhagen wound healing center in 1999/2000 and in 2011/2012. J Diabetes Res 2019;2019:6429575.

**Table S2** Antibodies used for immunofluorescence evaluation

| **Antibody** | **Host species** | **Dilution** | **Supplier (Catalogue number)** |
| --- | --- | --- | --- |
| **Primary antibodies** | | | |
| anti-HIF‑1α | mouse | 1:100 | BD Biosciences (610958) |
| Alexa Fluor® 594-anti-CD31 | mouse | 1:50 | BioLegend (303126) |
| anti-human Ki67 | rabbit | 1:200 | Abcam (ab15580) |
| **Secondary antibodies** | | | |
| FITC-anti-mouse Ig | goat | 1:500 | BD Biosciences (554001) |
| Alexa Fluor® 594-anti-rabbit IgG | donkey | 1:500 | Biolegend (406418) |

**Table S3** Primers

| **Primer** | **Sequence** | **Supplier** |
| --- | --- | --- |
| HIF‑1 | Forward: ACC CTA ACT AGC CGA GGA AGA  Reverse: GCA CCA AGC AGG TCA TAG GT | Thermo Fisher |
| VEGF | Forward: ACA TCA CCA TGC AGA TTA TGC G  Reverse: CGT TTT TGC CCC TTT CCC TT | Thermo Fisher |
| Actin | Forward: CGT ACA GGT CTT TGC GGA TG  Reverse: CCC TGG AGA AGA GCT ACG AG | Thermo Fisher |

**Table S4.** ABCB5^+^ MSC product release data

| **Date of production** | **Patient** | **Responder** | **Lot** | **Vitality^a^** | **Viability^b,c^** | **ABCB5^+^ cell content^c,d^** | **CD90^+^ cell content^c,e^** | **Potency^c^** | | | | | |
| --- | --- | --- | --- | --- | --- | --- | --- | --- | --- | --- | --- | --- | --- |
|  |  |  |  |  |  |  |  | **Angiogenesis** | **Endothelial differentiation** | | | **Immuno-modulation** | |
|  |  |  |  |  |  |  |  | **VEGF secretion^f^** | **Tube formation^g^** | | | **IL‑1RA secretion^h^** | |
| dd/mm/yyyy | # | Y/N | # | % of cells | % of cells | % of cells | % of cells | pg/ml | Category^i^ | | | pg/ml | Ratio stim / unstim |
|  |  |  |  |  |  |  |  |  | Well 1^k^ | | Well 2^l^ |  |  |
| 16/01/2018 | 02-001 | Y | C-AL04000-02 | 98.1 | 99.4 | 97.2 | 99.6 | 600 | 1.5 | | 2 | 5,112 | 3.2 |
| 10/04/2018 | 02-005 | Y | C-AL05000-02 | 97.7 | 99.9 | 93.8 | 99.8 | 396 | 1 | | 2 | 3,931 | 2.4 |
| 03/07/2018 | 02-009 | Y | C-AL05000-05 | 98.7 | 96.6 | 90.0 | 99.6 | 287 | 2 | | 2 | 3,859 | 2.3 |
| 17/07/2018 | 02-010 | Y | C-AL05000-06 | 98.7 | 96.6 | 90.0 | 99.6 | 287 | 2 | | 2 | 3,859 | 2.3 |
| 05/09/2018 | 02-010 | Y | C-AL05000-09 | 96.3 | 99.5 | 98.3 | 99.6 | 512 | 1 | | 2 | 7,730 | 2.7 |
| 16/10/2018 | 03-002 | Y | C-AL04000-03 | 98.4 | 99.9 | 98.3 | 99.8 | 835 | 1 | | 1 | 5,338 | 3.5 |
| 28/11/2018 | 03-002 | Y | C-AL04000-07 | 98.5 | 99.1 | 96.0 | 99.7 | 274 | 2 | | 2 | 10,130 | 2.9 |
| 05/12/2018 | 02-014 | N | C-AL05000-16 | 96.0 | 99.3 | 93.6 | 98.9 | 650 | 1 | | 2 | 8,550 | 3.0 |
| 15/01/2019 | 02-014 | N | C-AL04000-08 | 97.0 | 99.7 | 98.1 | 99.7 | 555 | 1.5 | | 1.5 | 4,869 | 3.2 |
| 22/01/2019 | 02-015 | Y | C-AL05000-20 | 97.0 | 99.0 | 98.6 | 99.7 | 421 | 2 | | 2 | 3,835 | 2.0 |
| 12/02/2019 | 04-003 | N | C-AL04000-10 | 97.5 | 95.6 | 98.9 | 97.2 | 782 | 1 | | 1 | 4,802 | 3.3 |
| 12/03/2019 | 02-015 | Y | C-AL04000-11 | 94.5 | 99.9 | 97.5 | 100 | 336 | 2 | | 2 | 3,483 | 3.0 |
| 26/03/2019 | 04-003 | N | C-AL05000-23 | 97.5 | 99.0 | 98.6 | 99.7 | 421 | 2 | | 2 | 3,835 | 2.0 |
| 02/04/2019 | 02-018 | Y | C-AL04000-12 | 97.5 | 99.9 | 98.3 | 99.8 | 591 | 1 | | 1 | 5,338 | 3.5 |
| 14/05/2019 | 02-018 | Y | C-AL05000-25 | 94.0 | 99.0 | 98.6 | 99.7 | 421 | 2 | | 2 | 3,835 | 2.0 |
| 21/05/2019 | 06-003 | N | C-AL04000-13 | 98.0 | 99.7 | 96.0 | 99.9 | 583 | 2 | | 2 | 5,535 | 2.2 |
| 21/05/2019 | 06-004 | N | C-AL05000-27 | 97.5 | 99.9 | 93.8 | 99.8 | 396 | 1 | | 2 | 3,931 | 2.4 |
| 23/05/2019 | 05-003 | Y | C-AL08000-04 | 98.5 | 99.1 | 99.2 | 99.5 | 701 | 3 | | 2 | 2,880 | 2.4 |
| 28/05/2019 | 02-020 | Y | C-AL04000-15 | 97.5 | 99.4 | 96.7 | 99.9 | 949 | 2 | | 2 | 6,126 | 2.3 |
| 05/06/2019 | 01-001 | Y | C-AL04000-17 | 97.0 | 99.7 | 96.0 | 99.9 | 583 | 2 | | 2 | 5,535 | 2.2 |
| 02/07/2019 | 06-005 | Y | C-AL05000-30 | 97.0 | 98.6 | 96.6 | 98.6 | 725 | 2 | | 1 | 16,430 | 6.5 |
| 02/07/2019 | 06-004 | N | C-AL04000-18 | 98.0 | 99.7 | 96.0 | 99.9 | 583 | 2 | | 2 | 5,535 | 2.2 |
| 03/07/2019 | 06-003 | N | C-AL04000-19 | 97.0 | 99.1 | 96.0 | 99.7 | 274 | 2 | | 2 | 10,130 | 2.9 |
| 09/07/2019 | 02-020 | Y | C-AL08000-10 | 97.0 | 98.1 | 99.0 | 98.0 | 502 | 2 | | 1 | 52,520 | 4.8 |
| 10/07/2019 | 05-003 | Y | C-AL04000-20 | 97.0 | 99.7 | 96.0 | 99.9 | 583 | 2 | | 2 | 5,535 | 2.2 |
| 13/08/2019 | 06-005 | Y | C-AL07000-06 | 97.0 | 99.8 | 97.4 | 99.5 | 1,034 | 3 | | 3 | 5,370 | 3.0 |
| 25/09/2019 | 04-007 | N | C-AL07000-15 | 93.5 | 99.4 | 97.6 | 99.5 | 853 | 2 | | 2 | 7,820 | 3.4 |
| 09/10/2019 | 04-008 | N | C-AL05000-37 | 93.5 | 99.3 | 94.3 | 99.4 | 412 | 2 | | 2 | 16,180 | 6.0 |
| 29/10/2019 | 02-021 | Y | C-AL05000-40 | 95.0 | 99.3 | 94.3 | 99.4 | 412 | 2 | | 2 | 16,180 | 6.0 |
| 05/11/2019 | 04-007 | N | C-AL10000-06 | 95.5 | 99.7 | 98.9 | 99.8 | 1,086 | 2 | | 2 | 8,680 | 2.2 |
| 19/11/2019 | 04-008 | N | C-AL07000-20 | 91.5 | 99.8 | 97.4 | 99.3 | 782 | 3 | | 2 | 6070 | 3.3 |
| 10/12/2019 | 04-011 | Y | C-AL07000-22 | 96.1 | 99.5 | 97.2 | 98.5 | 1,174 | 2 | | 2 | 7,340 | 3.3 |
| 13/01/2020 | 04-013 | Y | C-AL07000-25 | 95.1 | 99.7 | 97.1 | 99.8 | 432 | 2 | | 2 | 12,270 | 3.9 |
| 14/01/2020 | 02-021 | Y | C-AL07000-26 | 92.8 | 99.7 | 97.1 | 99.8 | 432 | 2 | | 2 | 12,270 | 3.9 |
| 21/01/2020 | 02-024 | Y | C-AL07000-28 | 96.2 | 99.2 | 99.6 | 99.4 | 1,589 | 3 | | 3 | 7,960 | 2.3 |
| 29/01/2020 | 08-001 | Y | C-AL07000-29 | 94.1 | 99.5 | 97.2 | 98.5 | 1,174 | 2 | | 2 | 7,340 | 3.3 |
| 25/02/2020 | 04-013 | Y | C-AL08000-18 | 98.3 | 99.1 | 99.2 | 99.5 | 701 | 3 | | 2 | 2,880 | 2.4 |
| 26/02/2020 | 08-002 | Y | C-AL07000-31 | 94.1 | 99.4 | 97.6 | 99.5 | 853 | 2 | | 2 | 7,820 | 3.4 |
| 03/03/2020 | 02-024 | Y | C-AL05000-48 | 94.1 | 98.6 | 98.4 | 98.6 | 725 | 2 | | 1 | 16,430 | 6.5 |
| **Specification** | | | | **≥ 75%** | **≥ 90%** | **≥ 90%** | **≥ 90%** | **> 46.9** | **≤3 in at least one well** | | | **> 125** | **> 1** |
| **% of products fulfilling the specification** | | | | **100%** | **100%** | **100%** | **100%** | **100%** | **100%** | | | **100%** | **100%** |
| **Mean (SD) all products** | | | | 96.4 (1.8) | 99.2 (0.9) | 96.8 (2.3) | 99.4 (0.6) | 541 (268) | 1.92 (0.57) | 1.88 (0.45) | | 2,828 (2052) | 3.2 (1.2) |
| **Mean (SD) products applicated to responders** | | | | 96.5 (1.7) | 99.2 (0.9) | 96.9 (2.4) | 99.5 (0.5) | 509 (281) | 1.98 (0.56) | 1.89 (0.51) | | 2,816 (2,089) | 3.3 (1.3) |
| **Mean (SD) products applicated to non-responders** | | | | 96.0 (2.1) | 99.2 (1.2) | 96.6 (1.9) | 99.4 (0.8) | 615 (230) | 1.79 (0.58) | 1.88 (0.31) | | 2,854 (2,057) | 2.9 (1.1) |

For more detailed descriptions of the release testing algorithm and the methods see Ballikaya S, Sadeghi S, Niebergall-Roth E, Nimtz L, Frindert J, Norrick A, et al. Process data of allogeneic *ex vivo*-expanded ABCB5^+^ mesenchymal stromal cells for human use: off-the-shelf GMP-manufactured donor-independent ATMP. Stem Cell Research & Therapy. 2020;11:482
^a^Defined as percentage of live cells determined by flow cytometry as propidium iodide-excluding cells
^b^Defined as percentage of metabolically active cells determined by flow cytometry as cells converting calcein acetoxymethylester to calcein
^c^Values were adopted from drug substance release testing; transferability of the results from drug substance release testing onto the final drug product was demonstrated in stability studies and method validation (Ballikaya et al., 2020)
^d^Determined by flow cytometry after incubation with an Alexa Fluor® 647-conjugated donkey anti-mouse IgG (H+L) secondary antibody (Thermo Fisher A‑31571) targeting the anti-ABCB5 antibody used for cell isolation
^e^Determined by flow cytometry after incubation with an Alexa Fluor® 647-conjugated mouse anti-human CD90 antibody (BioLegend 328116)
^f^Determined as VEGF concentration in the cell culture supernatant after 48 h culture under hypoxic conditions using the Invitrogen VEGF Human ELISA kit (Thermo Fisher) to evaluate the angiogenic potency of the ABCB5^+^ MSCs
^g^Determined by light microscopy after 19-22 h incubation on Geltrex® extracellular matrix gel (Thermo Fisher) to evaluate the endothelial trans-differentiation capacity of the ABCB5^+^ MSCs. HUVECs and human skin malignant melanoma cells served as positive and negative control, respectively
^h^Determined as IL‑1RA concentration in the cell culture supernatant after 48 h cocultivation with M1-polarized macrophages using the Qantikine® sandwich ELISA kit (R&D Systems) to evaluate the immunomodulatory potency of the ABCB5^+^ MSCs
^i^1, tubular branches of several cells forming a defined network-like structure; 2, tubular branches of several cells clustering together forming broad strands, formation of syncytia, areas of high cellular density lacking formation of tubular branches; 3, cells clustering together, building nodes and forming tubular branches that connect the nodes with each other; 4, only sporadic cells form tubular branches, partial node formation, but no or nearly no connections between nodes, no or only sporadic apoptotic cells; 5, largely apoptotic cells, no or only sporadic tubular branches; 6, no tubular branches
^k^Seeded cell concentration 1×10^5^/ml
^l^Seeded cell concentration 1.5×10^5^/ml

**Table S5.** LDPI measurements in the non-ischemic (right) and ischemic (left) hindlimb of OF1 mice with surgically induced hindlimb ischemia treated with vehicle or 5×10^6^ ABCB5^+^ MSCs

| **Day** | **Vehicle group** | | | | **MSC group** | | | |
| --- | --- | --- | --- | --- | --- | --- | --- | --- |
|  | **Animal ID** | **Right** (PU) | **Left** (PU) | **Ratio L/R**  (%) | **Animal ID** | **Right** (PU) | **Left** (PU) | **Ratio L/R**  (%) |
| **1 pre surgery** | 61 | 114.24 | 99.92 | 87.5 | 71 | 97.05 | 84.36 | 86.9 |
|  | 62 | 80.72 | 72.19 | 89.4 | 72 | 80.71 | 78.88 | 97.7 |
|  | 63 | 72.53 | 77.03 | 106.2 | 73 | 85.69 | 76.12 | 88.8 |
|  | 64 | 78.40 | 78.31 | 99.9 | 74 | 82.85 | 78.21 | 94.4 |
|  | 65 | 69.11 | 82.10 | 118.8 | 75 | 82.64 | 75.30 | 91.1 |
|  | 66 | 107.47 | 106.08 | 98.7 | 76 | 103.05 | 109.71 | 106.5 |
|  | 67 | 72.34 | 76.22 | 105.4 | 77 | 85.68 | 96.88 | 113.1 |
|  | 68 | 67.09 | 75.98 | 113.3 | 78 | 70.71 | 56.83 | 80.4 |
|  | 69 | 139.92 | 117.52 | 84.0 | 79 | 77.89 | 72.70 | 93.3 |
|  | 70 | 76.71 | 83.47 | 108.8 | 80 | 88.07 | 88.86 | 100.9 |
|  | **Mean** | **87.9** | **86.9** | **101.2** | **Mean** | **85.4** | **81.8** | **95.3** |
|  | **SD** | **24.3** | **15.4** | **11.5** | **SD** | **9.2** | **14.4** | **9.6** |
|  | **n** | **10** | **10** | **10** | **n** | **10** | **10** | **10** |
| **1 post surgery** | 61 | 118.89 | 29.90 | 25.2 | 71 | 85.99 | 33.68 | 39.2 |
|  | 62 | 83.92 | 26.04 | 31.0 | 72 | 96.69 | 29.94 | 31.0 |
|  | 63 | 96.25 | 26.71 | 27.8 | 73 | 102.69 | 46.08 | 44.9 |
|  | 64 | 113.30 | 33.01 | 29.1 | 74 | 66.93 | 22.44 | 33.5 |
|  | 65 | 62.56 | 25.36 | 40.5 | 75 | 84.15 | 31.78 | 37.8 |
|  | 66 | 115.28 | 47.02 | 40.8 | 76 | 97.55 | 37.75 | 38.7 |
|  | 67 | 80.09 | 48.48 | 60.5 | 77 | 99.97 | 41.69 | 41.7 |
|  | 68 | 88.11 | 27.31 | 31.0 | 78 | 98.27 | 37.78 | 38.4 |
|  | 69 | 75.57 | 43.47 | 57.5 | 79 | 74.80 | 43.30 | 57.9 |
|  | 70 | 102.68 | 46.29 | 45.1 | 80 | 102.09 | 31.84 | 31.2 |
|  | **Mean** | **93.7** | **35.4** | **38.9** | **Mean** | **90.9** | **35.6** | **39.4** |
|  | **SD** | **18.8** | **9.7** | **12.4** | **SD** | **12.4** | **7.1** | **7.8** |
|  | **n** | **10** | **10** | **10** | **n** | **10** | **10** | **10** |
| **3** | 61 | 86.42 | 37.70 | 43.6 | 71 | 65.35 | 32.22 | 49.3 |
|  | 62 | 75.31 | 38.11 | 50.6 | 72 | 77.57 | 58.58 | 75.5 |
|  | 63 | 76.26 | 53.17 | 69.7 | 73 | 68.05 | 45.91 | 67.5 |
|  | 64 | 82.39 | 26.60 | 32.3 | 75 | 76.48 | 56.73 | 74.2 |
|  | 65 | 67.67 | 39.46 | 58.3 | 76 | 75.68 | 66.95 | 88.5 |
|  | 66 | 70.55 | 34.55 | 49.0 | 77 | 91.86 | 50.88 | 55.4 |
|  | 68 | 88.40 | 34.96 | 39.6 | 78 | 69.98 | 55.56 | 79.4 |
|  | 69 | 129.88 | 71.64 | 55.2 | 79 | 73.82 | 39.95 | 54.1 |
|  | 70 | 77.16 | 52.81 | 68.4 | 80 | 47.69 | 11.41 | 23.9 |
|  | **Mean** | **83.8** | **43.2** | **51.9** | **Mean** | **71.8** | **46.5** | **63.1** |
|  | **SD** | **18.6** | **13.6** | **12.5** | **SD** | **11.8** | **16.8** | **19.6** |
|  | **n** | **9** | **9** | **9** | **n** | **9** | **9** | **9** |
| **5** | 61 | 65.81 | 46.85 | 71.2 | 71 | 82.56 | 59.64 | 72.2 |
|  | 62 | 73.97 | 38.27 | 51.7 | 72 | 73.29 | 73.06 | 99.7 |
|  | 63 | 69.90 | 69.62 | 99.6 | 73 | 81.06 | 87.08 | 107.4 |
|  | 65 | 61.48 | 52.34 | 85.1 | 75 | 108.37 | 72.39 | 66.8 |
|  | 66 | 82.89 | 33.68 | 40.6 | 76 | 80.53 | 73.56 | 91.3 |
|  | 68 | 71.53 | 24.33 | 34.0 | 77 | 103.17 | 64.67 | 62.7 |
|  | 69 | 66.95 | 31.44 | 47.0 | 78 | 104.30 | 116.85 | 112.0 |
|  | 70 | 59.33 | 56.88 | 95.9 | 79 | 67.65 | 44.85 | 66.3 |
|  | **Mean** | **69.0** | **44.2** | **65.6** | **Mean** | **87.6** | **74.0** | **84.8** |
|  | **SD** | **7.5** | **15.0** | **25.8** | **SD** | **15.5** | **21.2** | **20.1** |
|  | **n** | **8** | **8** | **8** | **n** | **8** | **8** | **8** |
| **7** | 62 | 96.95 | 80.22 | 82.7 | 71 | 116.25 | 93.48 | 80.4 |
|  | 63 | 69.81 | 82.63 | 118.4 | 72 | 76.92 | 66.52 | 86.5 |
|  | 65 | 78.32 | 70.31 | 89.8 | 73 | 82.40 | 98.43 | 119.5 |
|  | 66 | 91.88 | 65.66 | 71.5 | 75 | 72.07 | 51.72 | 71.8 |
|  | 68 | 76.64 | 21.96 | 25.7 | 76 | 86.41 | 77.92 | 90.2 |
|  | 69 | 109.39 | 77.50 | 70.9 | 77 | 111.90 | 97.46 | 87.1 |
|  | 70 | 82.40 | 57.92 | 70.3 | 78 | 101.49 | 127.68 | 125.8 |
|  |  |  |  |  | 79 | 86.62 | 92.75 | 107.1 |
|  | **Mean** | **86.5** | **65.2** | **75.6** | **Mean** | **91.8** | **88.2** | **96.1** |
|  | **SD** | **13.7** | **20.9** | **27.8** | **SD** | **16.3** | **23.0** | **19.3** |
|  | **n** | **7** | **7** | **7** | **n** | **8** | **8** | **8** |
| **14** | 62 | 95.21 | 96.49 | 101.3 | 71 | 121.64 | 143.45 | 117.9 |
|  | 63 | 98.38 | 105.18 | 106.9 | 72 | 69.52 | 70.14 | 100.9 |
|  | 65 | 95.61 | 87.46 | 91.5 | 73 | 76.12 | 94.99 | 124.8 |
|  | 66 | 91.17 | 76.43 | 83.8 | 75 | 123.32 | 115.11 | 93.3 |
|  | 68 | 68.03 | 75.53 | 111.0 | 76 | 103.84 | 144.68 | 139.3 |
|  | 69 | 113.49 | 138.90 | 122.4 | 77 | 112.95 | 86.50 | 76.6 |
|  | 70 | 103.61 | 78.40 | 75.7 | 78 | 87.97 | 87.59 | 99.6 |
|  |  |  |  |  | 79 | 69.51 | 77.77 | 111.9 |
|  | **Mean** | **95.1** | **94.1** | **98.9** | **Mean** | **95.6** | **102.5** | **108.0** |
|  | **SD** | **14.0** | **22.7** | **16.3** | **SD** | **22.7** | **28.8** | **19.6** |
|  | **n** | **7** | **7** | **7** | **n** | **8** | **8** | **8** |
| **21** | 62 | 80.72 | 75.32 | 93.3 | 71 | 79.20 | 68.31 | 86.3 |
|  | 63 | 102.14 | 107.85 | 105.6 | 72 | 63.07 | 58.53 | 92.8 |
|  | 65 | 68.88 | 76.87 | 88.5 | 73 | 86.85 | 75.29 | 86.7 |
|  | 66 | 94.64 | 89.32 | 94.4 | 75 | 76.41 | 64.80 | 84.8 |
|  | 68 | 101.35 | 95.27 | 94.0 | 76 | 108.77 | 93.70 | 86.2 |
|  | 69 | 93.17 | 69.67 | 74.8 | 77 | 89.74 | 73.13 | 81.5 |
|  | 70 | 83.63 | 68.18 | 81.5 | 78 | 84.73 | 72.64 | 85.7 |
|  |  |  |  |  | 79 | 63.83 | 63.10 | 98.9 |
|  | **Mean** | **89.2** | **83.2** | **90.3** | **Mean** | **81.6** | **71.2** | **87.9** |
|  | **SD** | **12.1** | **14.7** | **10.0** | **SD** | **14.8** | **10.7** | **5.4** |
|  | **n** | **7** | **7** | **7** | **n** | **8** | **8** | **8** |

PU, perfusion units

**Table S6.** CD31 expression in the ischemic thigh muscle of OF1 mice with surgically induced hindlimb ischemia treated with vehicle or ABCB5^+^ MSCs

|  | **Vehicle** | | **ABCB5^+^ MSCs** | | | | | |
| --- | --- | --- | --- | --- | --- | --- | --- | --- |
|  |  |  | **5×10^5^** | | **1×10^6^** | | **5×10^6^** | |
|  | **Animal ID** | **CD31 score** | **Animal ID** | **CD31 score** | **Animal ID** | **CD31 score** | **Animal ID** | **CD31 score** |
|  | V1 | 2 | L1 | 2 | M1 | 2 | H1 | 3 |
|  | V2 | 1 | L2 | 1 | M2 | 1 | H2 | 2 |
|  | V3 | 1 | L3 | 2 | M3 | 2 | H3 | 3 |
|  | V4 | 1 | L4 | 2 | M4 | 2 | H4 | 2 |
|  | V5 | 2 | L5 | 2 | M5 | 2 | H5 | 3 |
|  | V6 | 1 | L6 | 2 | M6 | 2 | H6 | 2 |
|  | V7 | 2 | L7 | 3 | M7 | 3 | H7 | 2 |
|  | V8 | 2 | L8 | 1 | M8 | 2 | H8 | 2 |
|  | V9 | 1 | L9 | 3 | M9 | 1 | H9 | 2 |
|  | V10 | 1 | L10 | 2 | M10 | 2 | H10 | 2 |
|  | V11 | 1 | L11 | 1 | M11 | 2 | H11 | 1 |
|  | V12 | 1 | L12 | 2 | M12 | 3 | H12 | 2 |
| **Mean** |  | 1.33 |  | 1.92 |  | 2.00 |  | 2.17 |
| **SD** |  | 0.49 |  | 0.67 |  | 0.60 |  | 0.58 |
| **N** |  | 12 |  | 12 |  | 12 |  | 12 |

CD31 expression was evaluated immunohistochemically and rated semi-quantitatively as 0 = none, 1 = minimal, 2 = slight, and 3 = moderate

**Table S7** Absolute wound surface area reduction from baseline by visit

| **Visit at** | **Full analysis set** (N=23) | | **Per-protocol set** (N=20) | |
| --- | --- | --- | --- | --- |
|  | n | Difference from baseline [cm^2^] | n | Difference from baseline [cm^2^] |
| Week 2 | 22 | 1.1 (0.4–2.4) | 20 | 1.2 (0.5–2.6) |
| Week 4 | 23 | 1.1 (0.3–2.7) | 20 | 1.6 (0.5–2.8) |
| Week 6 | 19 | 1.1 (-0.2–2.1) | 16 | 1.4 (0.0–2.3) |
| Week 8 | 20 | 1.8 (0.5–2.5) | 18 | 2.0 (0.6–2.6) |
| Week 12 | 23 | 1.7 (0.3–2.8) | 20 | 2.0 (0.9–2.9) |

Shown are medians (interquartile range)

**Table S8** Patients with complete wound closure and with ≥30% wound surface area reduction by visit

| **Visit at** | **Complete wound closure** | | | | **≥30% wound surface area reduction** | | | |
| --- | --- | --- | --- | --- | --- | --- | --- | --- |
|  | **Full analysis set** (N=23) | | **Per-protocol set** (N=20) | | **Full analysis set** (N=23) | | **Per-protocol set** (N=20) | |
|  | n | Patients with complete wound closure n (%) | n | Patients with complete wound closure n (%) | n | Patients with 30% wound surface area reduction n (%) | n | Patients with 30% wound surface area reduction n (%) |
| Week 2 | 23 | 0 (0) | 20 | 0 (0) | 23 | 11 (48) | 20 | 10 (50) |
| Week 4 | 23 | 1 (4) | 20 | 1 (5) | 23 | 13 (57) | 20 | 13 (65) |
| Week 6 | 19 | 2 (11) | 16 | 2 (13) | 19 | 9 (47) | 16 | 9 (56) |
| Week 8 | 20 | 1^a^ (5) | 17 | 1^a^ (6) | 20 | 15 (75) | 17 | 14 (82) |
| Week 12 | 23 | 6 (26) | 20 | 6 (30) | 23 | 17 (74) | 20 | 17 (85) |
| Any time | 23 | 6 (26) | 20 | 6 (30) | 23 | 19 (83) | 20 | 18 (90) |

^a^One of the two patients who had presented with full wound closure at week 6 missed the week‑8 visit

**Table S9** Wound exudation by visit

| **Visit at** | **Full analysis set** (N=23) | | | | **Per-protocol set** (N=20) | | | |
| --- | --- | --- | --- | --- | --- | --- | --- | --- |
|  | **n** | No. (%) of patients | | | **n** | No. (%) of patients | | |
|  |  | Low | Moderate | High |  | Low | Moderate | High |
| Day 0 | 23 | 10 (44) | 11 (48) | 2 (9) | 20 | 7 (35) | 11 (55) | 2 (10) |
| Week 1 | 23 | 11 (48) | 12 (52) | – | 20 | 9 (45) | 11 (55) | – |
| Week 2 | 22 | 11 (50) | 11 (50) | – | 19 | 10 (53) | 9 (47) | – |
| Week 4 | 23 | 13 (57) | 8 (35) | 2 (9) | 20 | 10 (50) | 8 (40) | 2 (10) |
| Week 6 | 19 | 10 (53) | 8 (42) | 1 (5) | 16 | 8 (50) | 7 (44) | 1 (6) |
| Week 8 | 20 | 13 (65) | 6 (30) | 1 (5) | 17 | 11 (65) | 5 (29) | 1 (6) |
| Week 12 | 23 | 12 (52) | 10 (44) | 1 (4) | 20 | 10 (50) | 9 (45) | 1 (5) |

Wound exudation was rated as low (dry), moderate (moist) or high (wet) according to Romanelli M, Vowden K, Weir D. Exudate management made easy. 2010. https://www.woundsinternational.com/resources/details/exudate-management-made-easy. Accessed 20 Dec 2021

**Table S10** Pain score by visit (FAS)

| **Visit at** | **n** | **Score** |
| --- | --- | --- |
| Day 0 | 23 | 1 (0–3) |
| Day 1 | 23 | 1 (0–2) |
| Day 2 | 23 | 1 (0–3) |
| Week 1 | 23 | 0 (0–2) |
| Week 2 | 22 | 1 (0–3) |
| Week 4 | 23 | 1 (0–3) |
| Week 6 | 19 | 1 (0–2) |
| Week 8 | 20 | 1 (0–1.5) |
| Week 12 | 23 | 1 (0–2) |

Pain was rated using a 0–10 point numerical rating scale with 0 = no pain and 10 = worst pain imaginable; shown are medians (interquartile range)
FAS, Full analysis set (N=23)

**Table S11** Quality of life scores by visit (FAS)

| **Scale** | **Day 0** | **Week 4** | **Week 12** |
| --- | --- | --- | --- |
| **Short Form (36) Health Survey subscale scores** | | | |
| Subscales | | | |
| Physical functioning^a^ | 50 (25–75) | 50 (15–70) | 60 (25–80) |
| Role functioning (physical)^a^ | 25 (0–75) | 25 (0–100) | 25 (0–100) |
| Role functioning (emotional)^a^ | 100 (0–100) | 100 (0–100) | 100 (0–100) |
| Social functioning^a^ | 88 (50–100) | 75 (50–100) | 75 (63–100) |
| Mental health^a^ | 76 (56–88) | 80 (60–88) | 72 (52–84) |
| Bodily pain^a^ | 74 (41–100) | 74 (52–100) | 74 (54–100) |
| Vitality^a^ | 55 (40–70) | 60 (45–75) | 53 (45–65) |
| General health perceptions^a^ | 55 (35–72) | 52 (31–72) | 52 (40–67) |
| Health transition^b^ | 3 (3–4) | 3 (2–3) | 3 (2–3) |
| **Dermatology Life Quality Index** | | | |
| Summary score | 6 (1–12) | 6 (1–11) | 4 (0–10) |

Shown are medians (interquartile range)
FAS, Full analysis set (N=23)
^a^Transformed scale (0–100)
^b^Raw scale

**Table S12** Serious treatment-emergent adverse events (SAS)

| **MedDRA system organ class**  Preferred term | **Number (%) of patients** |
| --- | --- |
| **Cardiac disorders** | **1 (4)** |
| Myocardial infarction | 1 (4) |
| **Infections and infestations** | **5 (22)** |
| Abscess limb | 1 (4) |
| Infected skin ulcer | 3 (13) |
| Localized infection | 1 (4) |
| **Injury, poisoning and procedural complications** | **1 (4)** |
| Foot fracture | 1 (4) |
| **Metabolism and nutrition disorders** | **2 (9)** |
| Hyperglycaemia | 1 (4) |
| Hypoglycaemia | 1 (4) |
| **Nervous system disorders** | **1(4)** |
| Sciatica | 1 (4) |
| **Renal and urinary disorders** | **1 (4)** |
| Nephrolithiasis | 1 (4) |
| **Any event** | **10 (43)** |

None of these events was related to the study treatment
*MedDRA* Medical Dictionary for Regulatory Activities
SAS, Safety analysis set (N=23)

**Table S13** Vital signs (SAS)

| **Parameter** | **Baseline (day 0)** | **Change at week 12** |
| --- | --- | --- |
| Body temperature (°C) | 36.6 (0.5) | 0.2 (0.4) |
| Blood pressure (mmHg) | | |
| Systolic | 135 (17) | 7 (16) |
| Diastolic | 78 (14) | -1 (9) |
| Heart rate (bpm) | 75 (9) | 3 (9) |

Shown are means (SD)
SAS, Safety analysis set (N=23)

**Table S14** Changes in physical examination findings from screening visit

| **Patient^a^** | **Organ system** | **Visit at** | **Specification** |
| --- | --- | --- | --- |
| 1 | Skin | Week 12 | Target ulcer nearly closed |
| 2 | Cardiovascular system | Week 12 | Sinus rhythm with some extrasystoles |
|  | Skin | Week 12 | Onychomycosis improved |
| 3 | Skin | Week 12 | Target wound smaller |
| 4 | Skin | Week 6.1^b^ | Onychomycosis on toe 1 and 2 left foot improved |
|  |  |  | Sanguineous callus on toe 2 left foot: warm, red, 2‑cent coin-sized skin defect on tip of toe |
|  |  | Week 12 | Onychomycosis on toe 1 and 2 left foot further improved |
|  |  |  | Sanguineous callus on toe 2 left foot: skin defect on tip of toe smaller, no signs of infection |
|  |  |  | Target ulcer: size reduced, no signs of infection |
| 5 | Lung and thorax | Week 6.1^b^ | Bronchitic breath sound |
|  | Skin | Week 12 | Two blisters on sole of right foot (target foot) almost healed |
| 6 | Skin | Week 12 | Blood blister lateral right to target wound^c^ |
| 7 | Musculoskeletal system | Week 6.1 | Back pain; bruised ribs; pain in left hip^d^ |
| 8 | General condition | Week 6.1^b^ | Common cold^e^ |
| 9 | Cardiovascular system | Week 6.1^b^ | No arrhythmia |
|  |  | Week 12 | No arrhythmia |
| 10 | Eyes | Week 6.1^b^ | Conjunctivitis^f^ |
|  | Skin | Week 6.1^b^ | Dermatomycosis both armpits^g^ |

SAS, Safety analysis set (N=23)
^a^Patients presenting with changes in physical examinations, numbered consecutively
^b^Week‑6.1 visit was intended for the second cell application, scheduled 1–3 days after the week‑6 efficacy follow-up visit
^c^Documented as treatment-emergent adverse event (TEAE) not related to study treatment
^d^Documented as TEAEs (“back pain”, “contusion” and “arthralgia”) not related to study treatment
^e^Documented as TEAE (“nasopharyngitis”) not related to study treatment
^f^Documented as TEAE not related to study treatment
^g^Documented as TEAE (“fungal skin infection”) not related to study treatment

**Table S15** Controlled clinical trials evaluating the efficacy of cell-based adjunctive strategies to treat diabetic foot ulcers

| **Cells / Product** | | **DFU baseline characteristics** | | **Number of wounds**  N (study treatment vs. control) | **Follow-up**  Weeks | **Mean surface area reduction from baseline** | | **Complete wound closure** | | **Ref.** |
| --- | --- | --- | --- | --- | --- | --- | --- | --- | --- | --- |
|  |  | **Mean wound age**  Weeks (study treatment vs. control) | **Mean surface area**  cm^2^ (study treatment vs. control) |  |  | % Reduction (study treatment vs. control) | Signifi-cant? | % Patients (study treatment vs. control) | Signifi-cant? |  |
| **Mesenchymal stem cells (MSCs)** | | | | | | | | | | |
| Autologous cultured BM-MSCs | |  | 4.2 vs. 3.8 | 22 vs. 23 | 12 |  |  | 83 vs. 45 | yes | [1] |
| Allogeneic AT-MSCs | | 8 vs. 7 | 24.5 vs. 25.8 | 10 vs. 10 | 12 |  |  | 90 vs. 80 |  | [2] |
| Hydrogel sheet containing allogeneic AT-MSCs | | 7 vs. 10 | 2.0 vs. 2.8 | 30 vs. 29 | 12 |  | yes | 82 vs. 53 | no | [3] |
| **Autologous adipose tissue** | | | | | | | | | | |
| Lipoaspirate | | 41 vs. 49 | 3.1 vs. 6.4 | 6 vs. 6 | 12 | 55 vs. 47 | no | 33 vs. 16 | no | [4] |
| Adipose-derived stromal vascular fraction | | 13 vs. 13 | 4.3 vs. 4.0 | 26 vs. 26 | 8 |  |  | 100 vs. 62 | yes | [5] |
| **Activated autologous platelet-rich plasma (PRP)** | | | | | | | | | | |
| PRP gel | ITT |  | 4.0 vs. 3.2 | 40 vs. 32 | 12 |  |  | 33 v. 28 | no | [6] |
|  | PP |  | 3.4 vs. 3.6 | 19 vs. 21 |  |  |  | 68 vs. 43 | no |  |
|  | PP, adjusted for baseline wound size |  | 2.0 vs. 2.4 | 16 vs. 19 |  |  |  | 81 vs. 42 | yes |  |
| PRP gel | |  |  | 12 vs. 12 | 20 |  |  | 25 vs. 0 |  | [7] |
| PRP dressing | |  | 5.0 vs. 5.2 | 30 vs. 30 | 6 | 86 vs. 82 | no |  |  | [8] |
| PRP injection | |  | 15.2 vs. 14.5 | 40 vs. 40 | 8 | 97 vs. 96 | no | 95 vs. 78 | yes | [9] |
| PRP injection + gel | | 47 vs. 47 |  | 36 vs. 36 |  |  |  | 86 vs. 64 | yes | [10] |
| Bio-functionalized scaffold based on hyaluronic acid and PRP | | mostly leg ulcers (DFU, vascular), but also burn and traumatic wounds | | 182 vs. 182 | 11.4 | 98 vs. 88 | yes |  |  | [11] |
| Lipoaspirate + PRP autograft | | 54 vs. 49 | 1.6 vs. 6.4 | 6 vs. 6 | 12 | 31 vs. 47 | no | 33 vs. 16 | no | [4] |
| **Allogeneic platelets** | | | | | | | | | | |
| Blood bank platelet concentrate | | 12 vs. 10 | 5.7 vs. 10.1 | 52 vs 48 | 12 | 96 vs. 82 | yes | 79 vs. 46 | yes | [12] |
| **Autologous skin cells** | | | | | | | | | | |
| Non-cultured skin cells (Spray-on Skin^TM^) | |  | 11.0 vs. 15.3 | 24 vs 25 | 26 |  |  | 67 vs. 64 | no | [13] |
| **Skin allografts** | | | | | | | | | | |
| Split-thickness skin allograft (TheraSkin® | | 18 vs. 16 | 4.2 vs. 3.9 | 50 vs. 50 | 12 | 78 vs. 50 | yes | 76 vs. 36 | yes | [14] |
| **Allogeneic living cell-containing skin substitutes** | | | | | | | | | | |
| Skin substitute generated by culture of fibroblasts onto a polyglactin mesh scaffold (Dermagraft®) | | 41 vs. 67 | 2.3 vs. 2.5 | 130 vs. 115 | 12 |  |  | 30 vs. 18 | yes | [15] |
| Bilayered skin substitute: keratinocytes and fibroblasts in collagen matrix | Graftskin, Apligraf® | 49 vs. 48 | 3.0 vs. 2.8 | 112 vs. 96 | 12 |  |  | 56 vs. 38 | yes | [16] |
|  |  | 104 vs. 88 | 3.0 vs. 3.0 | 33 vs. 39 | 12 |  |  | 52 vs. 26 | yes | [17] |
|  |  |  | 2.7 vs. 3.1 | 35 vs. 35 | 12 |  |  | 73 vs. 51 | yes | [18] |
|  | OrCel^TM^ |  |  | 20 vs. 20 | 12 |  |  | 35 vs. 20 |  | [19] |
| Placental membrane composed of living cells, growth factor and extracellular matrix (Grafix®) | | 16 vs. 18 | 3.4 vs. 3.9 | 50 vs. 47 | 12 |  |  | 62 vs. 21 | yes | [20] |

AT-MSCs, Adipose tissue-derived mesenchymal stem cells; BM-MSCs, Bone marrow-derived mesenchymal stem cells; ITT, Intention-to-treat population; PP, Per-protocol set

**References**

1. Debin L, Youzhao J, Ziwen L, Xiaoyan L, Zhonghui Z, Bing C. Autologous transplantation of bone marrow mesenchymal stem cells on diabetic patients with lower limb ischemia. Journal of Medical Colleges of PLA. 2008;23:106-15.
2. Uzun E, Güney A, Gönen ZB, Özkul Y, Kafadar İ H, Günay M, et al. Intralesional allogeneic adipose-derived stem cells application in chronic diabetic foot ulcer: Phase I/2 safety study. Foot Ankle Surg. 2021;27:636-42.
3. Moon KC, Suh HS, Kim KB, Han SK, Young KW, Lee JW, et al. Potential of Allogeneic Adipose-Derived Stem Cell-Hydrogel Complex for Treating Diabetic Foot Ulcers. Diabetes. 2019;68:837-46.
4. Smith OJ, Leigh R, Kanapathy M, Macneal P, Jell G, Hachach-Haram N, et al. Fat grafting and platelet-rich plasma for the treatment of diabetic foot ulcers: A feasibility-randomised controlled trial. Int Wound J. 2020;17:1578-94.
5. Han SK, Kim HR, Kim WK. The treatment of diabetic foot ulcers with uncultured, processed lipoaspirate cells: a pilot study. Wound Repair Regen. 2010;18:342-8.
6. Driver VR, Hanft J, Fylling CP, Beriou JM. A prospective, randomized, controlled trial of autologous platelet-rich plasma gel for the treatment of diabetic foot ulcers. Ostomy Wound Manage. 2006;52:68-70, 2, 4 passim.
7. Elsaid A, El-Said M, Emile S, Youssef M, Khafagy W, Elshobaky A. Randomized Controlled Trial on Autologous Platelet-Rich Plasma Versus Saline Dressing in Treatment of Non-healing Diabetic Foot Ulcers. World J Surg. 2020;44:1294-301.
8. Gupta A, Channaveera C, Sethi S, Ranga S, Anand V. Efficacy of Intralesional Platelet-Rich Plasma in Diabetic Foot Ulcer. J Am Podiatr Med Assoc. 2021;111:7.
9. Hossam EM, Alserr AHK, Antonopoulos CN, Zaki A, Eldaly W. Autologous Platelet Rich Plasma Promotes the Healing of Non-Ischemic Diabetic Foot Ulcers. A Randomized Controlled Trial. Ann Vasc Surg. 2022;82:165-71.
10. Orban YA, Soliman MA, Hegab YH, Alkilany MM. Autologous platelet-rich plasma vs conventional dressing in the management of chronic diabetic foot ulcers. Wounds. 2022;33:36-42.
11. De Angelis B, D'Autilio M, Orlandi F, Pepe G, Garcovich S, Scioli MG, et al. Wound Healing: In Vitro and In Vivo Evaluation of a Bio-Functionalized Scaffold Based on Hyaluronic Acid and Platelet-Rich Plasma in Chronic Ulcers. J Clin Med. 2019;8:1486.
12. Jeong SH, Han SK, Kim WK. Treatment of diabetic foot ulcers using a blood bank platelet concentrate. Plast Reconstr Surg. 2010;125:944-52.
13. Manning L, Ferreira IB, Gittings P, Hiew J, Ryan E, Baba M, et al. Wound healing with "spray-on" autologous skin grafting (ReCell) compared with standard care in patients with large diabetes-related foot wounds: an open-label randomised controlled trial. Int Wound J. 2022;19:470-81.
14. Armstrong DG, Galiano RD, Orgill DP, Glat PM, Carter MJ, Di Domenico LA, et al. Multi-centre prospective randomised controlled clinical trial to evaluate a bioactive split thickness skin allograft vs standard of care in the treatment of diabetic foot ulcers. Int Wound J. 2022;19:932-44.
15. Marston WA, Hanft J, Norwood P, Pollak R. The efficacy and safety of Dermagraft in improving the healing of chronic diabetic foot ulcers: results of a prospective randomized trial. Diabetes Care. 2003;26:1701-5.
16. Veves A, Falanga V, Armstrong DG, Sabolinski ML. Graftskin, a human skin equivalent, is effective in the management of noninfected neuropathic diabetic foot ulcers: a prospective randomized multicenter clinical trial. Diabetes Care. 2001;24:290-5.
17. Edmonds M. Apligraf in the treatment of neuropathic diabetic foot ulcers. Int J Low Extrem Wounds. 2009;8:11-8.
18. Zelen CM, Serena TE, Gould L, Le L, Carter MJ, Keller J, et al. Treatment of chronic diabetic lower extremity ulcers with advanced therapies: a prospective, randomised, controlled, multi-centre comparative study examining clinical efficacy and cost. Int Wound J. 2016;13:272-82.
19. Lipkin S, Chaikof E, Isseroff Z, Silverstein P. Effectiveness of bilayered cellular matrix in healing of neuropathic diabetic foot ulcers: Results of a multicenter pilot trial. Wounds. 2003;15:230-6.
20. Lavery LA, Fulmer J, Shebetka KA, Regulski M, Vayser D, Fried D, et al. The efficacy and safety of Grafix(®) for the treatment of chronic diabetic foot ulcers: results of a multi-centre, controlled, randomised, blinded, clinical trial. Int Wound J. 2014;11:554-60.
