# Supplementary material for: Translational development of ABCB5+ dermal mesenchymal stem cells for therapeutic induction of angiogenesis in non-healing diabetic foot ulcers
Source: Stem Cell Res Ther. 2022 Sep 5;13:455. doi: 10.1186/s13287-022-03156-9 (PMC9444095; doi:10.1186/s13287-022-03156-9)
Supplement: Supplementary file 2 — Additional file 2. Methods S1. Determination of the wound surface area. [file 13287_2022_3156_MOESM2_ESM.docx]

**Methods S1 Determination of the wound surface area**

**1. Measurement and calculation of the visible wound surface area**

Visible wound floor and visible wound perimeter were determined by using computerized planimetry (PictZar® software) from standardized wound photographs. Wound depth was measured by using a wound measuring probe at five different measuring points (center, 12 o’clock, 3 o’clock, 6 o’clock, 9 o’clock) and the mean calculated.

The visible wound surface area was calculated as the sum of surfaces of the visible wound floor and the wound wall as

Visible area = Area of visible wound floor + Visible wound perimeter × Mean wound depth

**2. Measurement and calculation of the not visible wound surface area**

Undermining areas were mapped on the skin around the wound perimeter by means of a probe and a marker. The mapped areas were then measured by using computerized planimetry (PictZar® software) from standardized photographs and the values multiplied by 2.5 to obtain an estimation accounting for the floor, ceiling and wall portions for each undermining area.

The not visible wound surface area was calculated as the sum of surfaces of undermining areas as

Not visible area = Undermining area_1_ + Undermining area_2_ + … + Undermining area_X_

**3. Calculation of the total wound surface area**

Total wound surface area was calculated as

Total area = Visible area + Not visible area
