# Supplementary material for: Translational development of ABCB5+ dermal mesenchymal stem cells for therapeutic induction of angiogenesis in non-healing diabetic foot ulcers
Source: Stem Cell Res Ther. 2022 Sep 5;13:455. doi: 10.1186/s13287-022-03156-9 (PMC9444095; doi:10.1186/s13287-022-03156-9)
Supplement: Supplementary file 3 — Additional file 3. Figure S1. Validation of the rabbit anti-human/mouse CD31 antibody. Figure S2. Blood flow recovery following surgically indcued hindlimb ischemia in OF1 mice. [file 13287_2022_3156_MOESM3_ESM.docx]

**
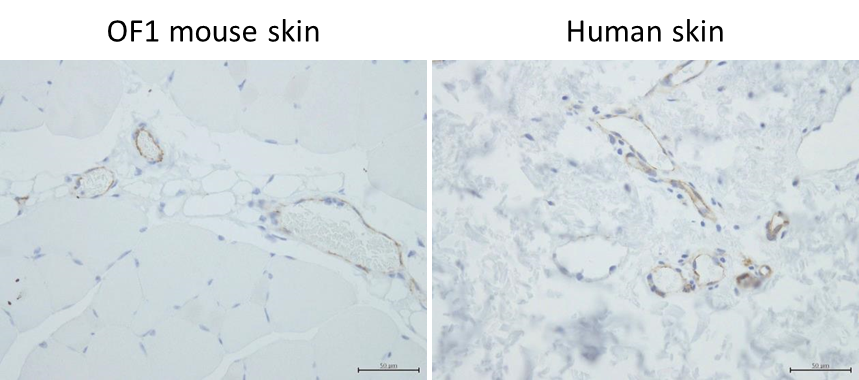
Figure S1.** Validation of the rabbit anti-human/mouse CD31 antibody (ab28364, Abcam) for immunohistochemical detection of CD31, showing cytoplasmic staining of OF1 mouse and human endothelium. Thus, the staining protocol was suitable to picture the formation of capillaries generated from both mice resident cells and administered human MSCs. Scale bars: 50 µm.


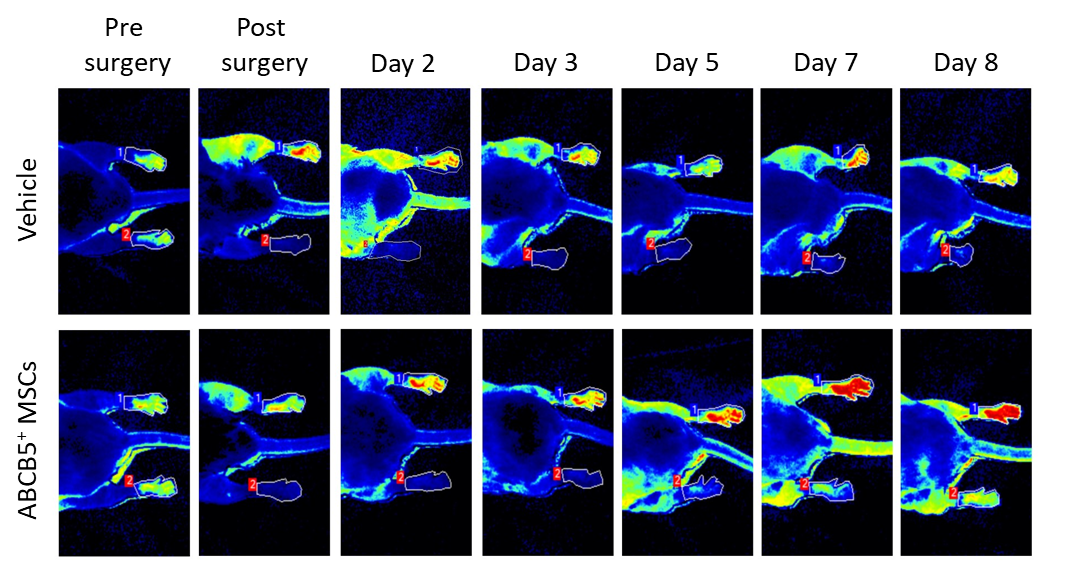


**Figure S2.** Blood flow recovery following surgically induced hindlimb ischemia in OF1 mice. Representative LDPI acquisitions from a mouse treated on day 0 with vehicle (upper row) or 5×10^6^ ABCB5^+^ MSCs (lower row) on day 2. The non-ischemic limbs are marked with “1”, the ischemic limbs with “2”.
